# Characterization of the SARS‐CoV‐2‐Specific T Cell Responses in Rheumatoid Arthritis Subjects Vaccinated for COVID‐19 Protection

**DOI:** 10.1002/eji.70105

**Published:** 2025-12-19

**Authors:** Jaeyoon Song, Ricardo da Silva Antunes, Mehrnaz Agili Seyede, Monica Guma, Alessandro Sette, Alessandra Franco

**Affiliations:** ^1^ School of Medicine Department of Pediatrics University of California San Diego La Jolla California USA; ^2^ Center For Vaccine Innovation, La Jolla Institute for Immunology (LJI); Department of Medicine, Division of Infectious Diseases and Global Public Health University of California San Diego (UCSD) La Jolla California USA; ^3^ Department of Medicine, School of Medicine University of California San Diego La Jolla California USA

**Keywords:** CD4^−^CD8^−^ double negative (DN) T cells, regulatory T cells, SARS‐CoV‐2 vaccination in rheumatoid arthritis, T cell memory

## Abstract

The efficacy of mRNA‐based vaccines to prevent COVID‐19 in autoimmune patients is controversial due to immunosuppressive therapies. Here, we characterized the T cell responses to SARS‐CoV‐2 in rheumatoid arthritis (RA) subjects, who received as few as one or up to seven vaccine injections. The study population had different disease severities and an association with other autoimmune comorbidities. All the subjects studied showed SARS‐CoV‐2 spike‐specific CD4+ T helper (Th) cells in circulation, and in most of the subjects, CD8^+^ cytotoxic T cells. CD4^+^ and CD8^+^ T cells were CCR6+, suggesting trafficking to tissues. T cell memory did not correlate with the number of vaccine boosts received. Nonspike‐specific T cells were enumerated in subjects who had symptomatic or asymptomatic COVID‐19. Spike‐ and nonspike‐specific regulatory T cells (Treg) were detectable in most subjects. CD4^−^ CD8^−^ double‐negative (DN) T cells expanded in response to SARS‐CoV‐2 peptides. DN T cells co‐cultured with autologous myeloid dendritic cells (DC) differentiated into CD8^+^ T cells, depending upon the strength of the stimuli. RA subjects responded to mRNA‐based vaccination for COVID‐19 protection, with no correlation with the number of injections. DN T cells differentiated into CD8^+^ T cells after stimulation, potentially exacerbating inflammation in RA vaccine recipients.

## Graphical abstract

SARS‐CoV‐2 vaccination in rheumatoid arthritis leads to the development of CD4^+^ and CD8^+^ spike‐specific T cell memory, regardless of immunosuppressive therapies, and the number of vaccine injections.

CD4^−^CD8^−^ double‐negative (DN) T cells responded to SARS‐CoV‐2 peptide epitopes and differentiated into CD8^+^ cytotoxic T cells after stimulation, suggesting a role in exacerbating the inflammation in RA subjects.

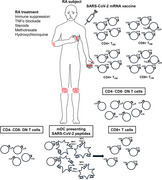

**Abbreviations**
CTL: cytotoxic T cells
DN: double‐negative T cells
T_CM_
: central memory T cells
T_EM_
: effector memory T cells
T_EMRA_
: effector memory cells re‐expressing CD45RA
Th: T helper cells
Treg: regulatory T cells

## 1 Introduction

In vaccine recipients for COVID‐19 protection, the humoral and cellular immune response with the Moderna mRNA 1273, Pfizer/BioNTech BNT162b2, and Janssen Ad26.COV2.S and Novavax NVX‐CoV2373 vaccines have been well described, with reports of the development of B‐cell and T‐cell memory [1, 2].

In autoimmunity, the chronic use of immunosuppressive medications raises the question of SARS‐CoV‐2 vaccine potency in affected subjects, depending on the antigenic targets and formulations. In rheumatoid arthritis (RA), a systemic autoimmune disease initiated by autoreactive T cells [3, 4], poor B‐cell and T‐cell responses against SARS‐CoV‐2 epitopes have been reported following mRNA COVID‐19 vaccination due to the chronic uptake of immunosuppressive therapies [5].

Here, we addressed SARS‐CoV‐2‐specific T cell responses and the development of T cell memory in RA subjects, selected for different clinical presentations and different ongoing immunosuppressive treatments. We characterized their virus‐specific CD4^+^ T helper (Th) cells and CD8^+^ cytotoxic T cells (CTL), their memory development, and explored the expansion and phenotype of SARS‐CoV‐2‐specific regulatory T cells (Treg).

Our study highlighted peculiarities within the T cell lineages in these patients involving CD8^+^ T cells that expressed the homing receptor CCR6, suggesting trafficking to tissues. Notably, we report the expansion of proinflammatory CD4^−^CD8^−^‐double‐negative (DN) T cells that polarized into single positive CD8^+^ CTL upon strong T cell receptor (TcR) signaling, which could potentially exacerbate the inflammatory process. Proinflammatory CD8^+^ that differentiate from DN T cells may be the cause of clinical relapses following repeated mRNA vaccination that have been reported in some autoimmune subjects [5, 6].

## 2 Materials and Methods

### 2.1 Study Population

RA patients have been enrolled at the Rheumatology clinic of the University of California San Diego (IRB# 191900).

Sixteen subjects, 15 females and 1 male, aged 40–78, participated in the study (Table 1). Twelve subjects were studied for T cell responses to SARS‐CoV‐2 (subjects 1–12), and four additional subjects (subjects 13–16) were enrolled to sort by flow cytometry DN CD4^−^CD8^−^ T cells and myeloid dendritic cells (DC). Ten subjects were Hispanic, five were Caucasians, and one was of Arabian/Caucasian mixed race.

**TABLE 1:** Characteristics of the RA subjects enrolled in the study.

Study#	Sex	Ethnicity	Age	Additional systemic autoimmunity	Organ‐specific autoimmunity	COVID19	Number of vaccinations	Therapy	Rheumatoid factors, autoantibodies
1	F	Hispanic	40	Sjogren	Diabetes	Yes	2	Adalimumab (TNF alpha blockade)	RF, ANA
2^a^	F	Caucasian	56	No	No	Yes	5	Sulfasalazine (Azulfidine), hydroxychloroquine	RF, ANCA
3	F	Caucasian	74	No	Hashimoto	No	5	no	RF
4	F	Hispanic	50	No	Hashimoto	Yes	2	Ibuprofen/Leflunomide	RF, anti‐cardiolipin
5	F	Arab/Caucasian	60	No	Hashimoto	Yes	2	no	RF, anti‐CCP
6	F	Hispanic	46	No	Hashimoto, Diabetes	Yes, our findings	2	Prednisone/Methotrexate/Naproxen	RF, ANA, anti‐CCP
7	F	Hispanic	54	Sjogren	No	Yes	2	Adalimumab/Prednisone/Etanercept/Methotrexate/Ibuprofen	ANA, anti‐Ro, anti‐CCP
8	F	Hispanic	46	No	No	Yes	4	Ibuprofen	anti‐CCP
9	F	Hispanic	48	No	No	Yes	4	Ibuprofen	ANA
10	F	Hispanic	60	No	No	Yes, our findings	1	Hydroxychloroquine/Methotrexate/Prednisone	ANA, anti‐Ro, anti‐ribonuclear protein, Smith antibody, anti‐C4
11	F	Hispanic	64	No	No	Yes, our findings	3	Ibuprofen/Leflunomide/Prednisone	Anti‐CCP, anti‐thyroperoxidase
12	F	Caucasian	78	SLE	Hashimoto	No	7	Etanercept/Methotrexate	RF, anti‐CCP
13	F	Hispanic	44	No	No	Yes	1	Methotrexate/Hydroxychloroquine/Ibuprofen	Not tested
14	M	Caucasian	49	No	No	Yes	2	Methotrexate/Hydroxychloroquine	RF, anti‐CCP
15	F	Caucasian	74	No	Hashimoto	Yes	5	Methotrexate	RF, anti‐CCP, anti‐neutrophilic cytoplasmic (ANCA), anti‐C3, anti‐C4
16	F	Hispanic	60	No	No	Yes	3	Upadacitinib/Ibuprofen	RF, anti‐CCP

^a^ This subject developed RA after COVID‐19 infection.

The cohort was chosen because of the variable numbers of mRNA‐based vaccines received, different clinical presentations, autoimmune comorbidities, and immunosuppressive therapeutic regimen (Table 1). Clinically, subjects 2, 8, 9, 10, 11, 13, 14, and 16 had only RA; subjects 1, 7, and 12 had systemic autoimmune comorbidities such as Sjogren syndrome, lupus and psoriasis; and subjects 1, 3, 4, 5, 6, 12, and 15 had organ‐specific autoimmunity (diabetes type 1 and Hashimoto thyroiditis) (Table 1).

Blood was drawn 1 to 2 years after the last vaccine injection. Eleven of sixteen reported previous symptomatic COVID‐19 infection. Subjects 6, 10, and 11, who reported no previous COVID‐19 symptoms, showed in our study T cell responses to the nonspike peptide epitopes, suggesting previous asymptomatic infection (Table 1).

Four healthy vaccinated subjects, all females, Caucasian, with ages between 26 and 55, served as a control for the enumeration and characterization of SARS‐CoV‐2‐specific regulatory T cells (Treg).

### 2.2 Peptides

CD4^+^ T helper (Th) and CD8^+^ cytotoxic (CTL) responses to the spike peptide epitopes have been studied with overlapping peptides, 15 amino acids long with 10 amino acid overlaps, spanning the entire protein (253 peptides). The protein sequence was based on Wuhan‐Hu‐1 isolates as reference (GenBank ID: MN908947) (Table S1). This antigenic approach has been extensively validated in acute and convalescent samples [7, 8, 9]. T cell responses to SARS‐CoV‐2 nonspike antigens have been measured by stimulating PBMC in vitro with two combined peptide pools (pool 1, 69 amino acids, and pool 2, 215 amino acids), spanning the nonspike regions of SARS‐CoV‐2. To design epitope pools with increased human leucocyte antigen (HLA) coverage and broad recognition by demographically and geographically diverse populations, experimentally defined epitopes from the nonspike (R) region of SARS‐CoV‐2 were selected based on our recent meta‐analysis [9]. Epitopes with higher sequence homology with common corona cold viruses (CCC) were not included (Table S2).

Peptides were synthesized as crude material (TC Peptide Lab, San Diego), resuspended in DMSO, pooled according to a mega pool design, and re‐lyophilized [10].

### 2.3 T Cell‐Activation‐Induced Marker (AIM) Assay

Peripheral blood mononuclear cells (PBMC) were separated by Ficoll Hypaque density centrifugation. After counting, 1 × 10^6^ cells were stimulated in 96‐well U‐bottom plates with 1 µg/mL of spike peptide pool and with the nonspike peptide pools (Tables S1 and S2).

PBMC cultured with 0.1% DMSO (solvent), the same concentration of DMSO in the peptide pool‐stimulated cultures, served as unstimulated controls. Cell cultures were harvested 24 h later and then stained with monoclonal antibodies and analyzed by flow cytometry to study T cell activation, CCR6 expression, and T cell memory phenotypes. The antibodies included: anti‐CD3‐AF700 (clone OKT3, mouse IgG2aκ, BioLegend), anti‐CD4‐BV605 (clone RPA‐T4, mouse IgG1κ, BD Bioscience), anti‐CD8‐PE/CF594 (clone RPA‐T8, mouse IgG1k, BD Bioscience), anti‐4‐1BB‐allophycocyanin (clone 4B4‐1, mouse IgG1κ, BioLegend), anti‐OX40‐PE/Cy7 (clone Ber‐ACT35, mouse IgG1κ, BioLegend), anti‐CD69‐PE (clone FN50, mouse IgG1κ, BD Bioscience), anti‐CCR6‐PerCp/Cy5.5 (clone 11A9, mouse IgG1κ, BD Bioscience), anti‐CD45RA‐APC‐H7 (clone HI100, mouse IgG2bk, BD Bioscience), and anti‐CCR7‐FITC (clone G043H7, mouse IgG2aκ, BioLegend). Data were recorded on BD Canto II and analyzed with FlowJo software version 10 (Tree Star). Isotype controls for each antibody were tested to set negative populations.

Antigen‐specific responses were determined by the expression of T cell activation‐induced cell markers (AIM) assay by measuring the co‐expression of 4‐1BB and OX40 on CD4^+^ T cells (two TNF family co‐stimulatory molecules which are up‐regulated following CD4^+^ T cell receptor signaling) and by measuring the co‐expression of 4‐1BB and CD69 on CD8^+^ T cells (early activation markers expressed in hematopoietic stem cells involved in lymphocyte homing and trafficking) [7].

T cell responses are presented as stimulation index (SI), defined as the percentage of T cells responding to spike and nonspike peptides, divided by the unstimulated control signal.

The expression of the chemokine receptor CCR6 on AIM^+^ CD4^+^ and CD8^+^ T cells was also analyzed. Terminally differentiated effector memory (T_EMRA_) (CD45RA^+^ CCR7^−^), effector memory (T_EM_) (CD45RA^−^ CCR7^−^), and central memory (T_CM_) (CD45RA^−^ CCR7^+^), were enumerated by gating on AIM^+^ CD4^+^ and CD8^+^ T cells.

Treg were defined by the co‐expression of CD4 (anti‐CD4‐BV605, clone RPA‐T4, mouse IgG1κ, BD Bioscience) and CD25^high^, with a mean fluorescence of approximately 10^4^ (anti‐CD25‐BV421, clone M‐A251, mouse IgG1κ, BD Bioscience). In some subjects, Treg were further phenotyped with a panel of functional markers, gating on FOXP3+ CD4+ CD25^high^ T cells, as described below.

### 2.4 Characterization of Regulatory T Cells

Intracellular FOXP3 expression was defined by staining with anti‐FOXP3‐PE (clone 259D, mouse IgG1κ, BioLegend), in combination with surface markers by using a cocktail of monoclonal antibodies: anti‐CD3‐AF700 (clone OKT3, mouse IgG2aκ, BioLegend), anti‐CD4‐PerCp/Cy5.5 (clone RPA‐T4, mouse IgG1κ, eBioscience), anti‐ICOS‐PE/Cy7 (clone ISA‐3, mouse IgG1κ, eBioscience), anti‐CD25‐BV421 (clone M‐A251, mouse IgG1κ, BD Biosciences), anti‐CD45RA‐APC‐H7 (clone HI100, mouse IgG2bκ, BD Biosciences), anti‐CD127 (IL‐7Ra)‐APC (clone eBioRDR5, mouse IgG1κ, eBioscience), anti‐PD‐1‐BV605 (clone EH12.2H7, mouse IgG1κ, BioLegend), and anti‐CTLA‐4‐PE/Dazzle594 (clone BNI3, mouse IgG2aκ, BioLegend). The surface expression of amphiregulin (AREG) was determined with a polyclonal goat anti‐human AREG IgG, and a secondary polyclonal donkey anti‐goat IgG‐NL493 antibody from R&D Systems.

### 2.5 Lymphokines

Lymphokine secretion by T cells was determined by ELISA in culture supernatants. Interleukin (IL)‐2 was measured with the Invitrogen Human IL‐2 ELISA kit and IFN‐γ with the BD Biosciences human IFN‐γ kit, according to the manufacturer's instructions. We customized our assay for the measurement of IL‐10 by purchasing recombinant human IL‐10 from BD Pharmingen, primary and secondary anti‐IL‐10 antibodies from BD Biosciences, and streptavidin HRP from BD Biosciences.

### 2.6 Characteristics of CD4^−^ CD8^−^ DN T Cells

The activation state and phenotype of DN T cells were further characterized by surface staining with the following monoclonal antibodies: anti‐CD3‐AF700 (clone OKT3, mouse IgG2aκ, BioLegend), anti‐CD4‐BV605 (clone RPA‐T4, mouse IgG1κ, BD Biosciences), anti‐CD8‐PE/CF594 (clone RPA‐T8, mouse IgG1κ, BD Biosciences), anti‐CD25‐BV421 (clone M‐A251, mouse IgG1κ, BD Biosciences), anti‐HLA‐DR‐APC‐H7 (clone G46‐6, mouse IgG2aκ, BD Biosciences), anti‐IL‐7Ra‐APC (clone eBioRDR5, mouse IgG1κ, eBioscience), anti‐PD‐1‐AF488 (clone EH12.2H7, mouse IgG1κ, BioLegend).

In separate experiments, CD4^−^ CD8^−^ DN T cells were sorted by flow cytometry after stimulation, using the same antibodies described for their characterization.

Cells were then co‐cultured with autologous myeloid dendritic cells (DC) to study their possible linear differentiation to CD4^+^ or CD8^+^ T cells.

Activated myeloid DC in PBMC were defined with a cocktail of monoclonal antibodies: anti‐CD14‐PE/Cy7 (clone M5E2, mouse IgG2aκ, BD Biosciences), anti‐CD11c‐APC (clone B‐Ly6, mouse IgG1κ, BD Biosciences), anti‐CD11b‐APC/Cy7 (clone ICRF44, mouse IgG1κ, BD Biosciences), and anti‐CD86‐FITC (clone 2331, mouse IgG1κ, BD Biosciences).

DN T cells were defined by using anti‐CD3‐AF700 (clone OKT3, mouse IgG2aκ, BioLegend), anti‐CD4‐BV605 (clone RPA‐T4, mouse IgG1κ, BD Biosciences), anti‐CD8‐PE/CF594 (clone RPA‐T8, mouse IgG1κ, BD Biosciences).

The FACS‐sorting strategies included gating on CD3^+^ and CD3**^−^** PBMC (Figure S1). To obtain DN T cells, gating on the CD3^+^ population, CD4^+^ and CD8^+^ T cells were excluded. To obtain myeloid DC, after gating on CD3**^−^** PBMC, the double‐positive CD11c^+^ and CD11b^+^ CD86^+^ were FACS‐sorted.

FACS‐sorted DN T cells alone, or those in co‐cultures with FACS‐sorted activated myeloid DC, were stimulated in vitro with agonistic antibodies to CD3 and CD28, anti‐CD3 (BD Bioscience, clone UCHT1), and anti‐CD28 (BD Bioscience, clone CD28.2). The cell ratios in these experiments were 1:4 DN T cells/DC (2.5 × 10^4^/10 × 10^4^).

### 2.7 Statistical Analysis

Data were analyzed using Prism software version 10 (GraphPad Software). To compare the percentage of AIM^+^ T cells in the unstimulated cultures and peptide‐stimulated cultures, we used a nonparametric paired test. A *p*‐value < 0.05 was considered significant.

## 3 Results

### 3.1 Characterization of SARS‐CoV‐2 Spike and Nonspike Specific CD4^+^ and CD8^+^ in RA Subjects

Twelve RA subjects, all females, aged 40–78, were enrolled in the study and provided blood samples to characterize SARS‐CoV‐2‐specific T cells. RA subjects received mRNA‐based vaccination for SARS‐CoV‐2 protection, with the number of injections ranging from 1 to 7, received within weeks to a few months before blood drawing. Two subjects never contracted COVID‐19, namely subjects 3 and 12, and 7 of 12 subjects reported symptomatic COVID‐19 infection, namely subjects 1, 2, 4, 5, 7, 8, and 9 (Table 1).

RA subjects were enrolled sequentially, regardless of the therapy to control inflammation.

PBMC were stimulated in vitro for 24 h with SARS‐CoV‐2 spike and nonspike peptide epitopes (sequences shown in Tables S1 and S2). T cell responses were measured by flow cytometry using the AIM assay. This antigenic design and T cell read‐out were extensively validated in COVID‐19 acute and convalescent samples, and healthy vaccine recipients by our collaborators [7, 8, 9, 11].

**FIGURE 1:**
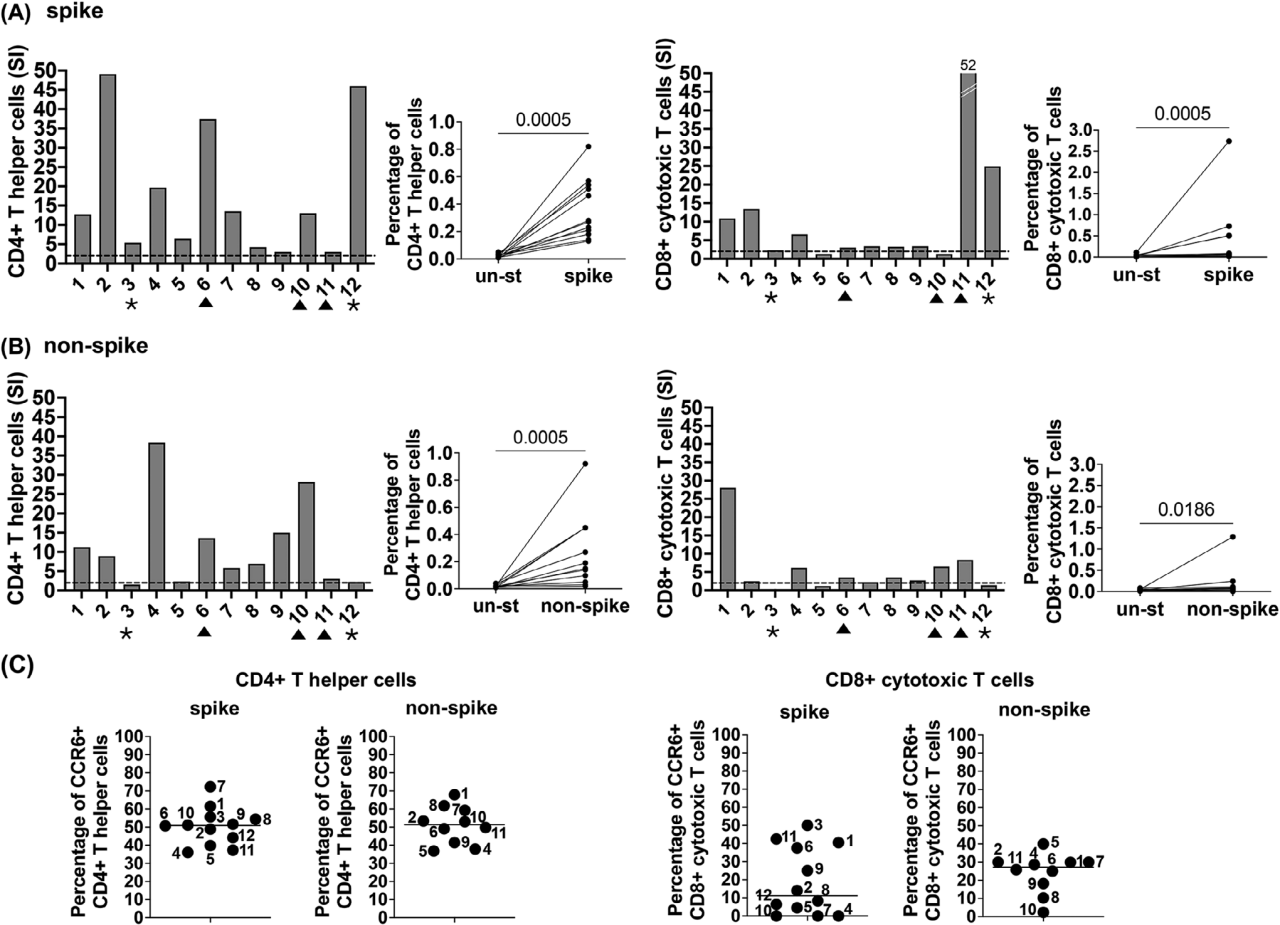
Characterization of SARS‐CoV‐2 spike‐ and nonspike‐specific CD4^+^ and CD8^+^ T cells. PBMC were separated from twelve vaccinated RA subjects who received different numbers of mRNA vaccine injections for COVID‐19 protection. Three of twelve showed a T cell response to the nonspike peptide pools, suggesting a previous asymptomatic COVID‐19 infection (marked with a triangle within the X axis). Subjects 3 and 12 never contracted COVID‐19 (marked with an asterisk in the X axis). (A) CD4^+^ Th and CD8^+^ cytotoxic T cell responses to the spike peptide pools. Left panels: after first gating on CD3^+^ T cells, CD4^+^ Th were studied with anti‐CD4, anti‐4‐1BB, and anti‐OX40 antibodies. The percentage of AIM^+^ CD4+ Th cells from unstimulated control and SARS‐CoV‐2 CD4 spike‐stimulated cells is presented as the stimulation index (SI). We considered a positive response as an SI > 2, cut off marked with a dotted line in the graphs. Antigen‐specific T cell responses were compared with the Wilcoxon matched‐pairs signed‐rank test. Right panels: CD8^+^ T cell response to the spike peptide pool. The activation of CD8^+^ T cells was studied by first gating on CD3^+^ T cells, then gating on CD8+ T cells within the 4‐1BB and CD69 double‐positive population. The percentage of AIM^+^ CD8^+^ CTL from unstimulated control and SARS‐CoV‐2 spike‐stimulated cells, presented as the stimulation index (SI). We considered a positive response SI > 2, cut off marked with a dotted line in the graphs. Antigen‐specific T cell responses were also compared by the Wilcoxon matched‐pairs signed‐rank test. (B) CD4^+^ Th responses (left panels) and CD8^+^ cytotoxic T cell responses (right panels) to the nonspike peptide pools presented as SI. (C) CCR6 expression on CD4^+^ OX40^+^, 4‐1BB^+^, median % 50.9. CCR6 was also high in nonspike‐specific CD4^+^ Th cells, median % 51.4. CD8^+^ CTL expressed CCR6, although to a lesser extent than CD4^+^ Th cells, median % 11:2 for spike‐specific T cells, and median % 27.2 for nonspike‐specific T cells.

All the subjects showed a CD4^+^ Th response to the spike peptide pool (Figure 1A, left). Ten of twelve subjects had both CD4^+^Th and CD8^+^ CTL responses to the spike peptide pool, namely 1, 2, 3, 4, 6, 7, 8, 9, 11, and 12. Subjects 5 and 10 had a very low or undetectable CD8^+^ CTL response (Figure 1A, right graph). Overall, comparison of unstimulated PBMC with spike‐stimulated PBMC showed a significant difference in the percentages of CD4^+^ and CD8^+^ T cells, as analyzed by the Wilcoxon matched‐pairs signed‐rank test (Figure 1A, middle graph).

Several subjects in the cohort reported symptomatic COVID‐19. In PBMC derived from these subjects, namely 1, 2, 4, 5, 7, 8, and 9 (Table 1), we found different patterns of T cell recognition of the nonspike peptide epitopes. Nine subjects, 1, 2, 4, 6, 7, 8, 9, 10, and 11, showed CD4^+^ Th and CD8^+^ T cell expansion. Subjects 5 and 12 showed only a CD4^+^ Th response, but not a CD8^+^ T cell response (Figure 1B).

A few of the subjects (3, 6, 10, 11, and 12) did not report a previous COVID‐19 infection. Of interest, subjects 6, 10, and 11 did show CD4^+^ Th and CD8^+^ CTL responses to the nonspike peptide pool (Figure 1B), suggesting that they previously experienced an asymptomatic COVID‐19 infection, as indicated in a previous report (14).

The expression of the chemokine receptor CCR6, which determines homing to the endovascular endothelial compartment, lungs, gut, neurons, myometrium, and vaginal epithelial cells, was also evaluated on AIM^+^ T cells. CCR6 was expressed on spike‐ and nonspike‐specific CD4^+^ Th cells (median % 50.9 and 51.4, respectively), as well as on spike‐ and nonspike‐specific CD8^+^ T cells (median % 11.2 and 27.2, respectively) (Figure 1C). The results suggested a similar tissue distribution for the spike and nonspike‐specific CD4^+^ and CD8^+^ T cells.

The expression of CCR6 on CD8^+^ T cells was unique in this cohort of patients, differing from our data previously reported on healthy vaccinated subjects, where only CD4^+^ Th cells were found to be CCR6^+^ [12].

### 3.2 Characterization of SARS‐CoV‐2‐Specific CD4^+^ and CD8^+^ Memory T Cells in RA Subjects

**FIGURE 2:**
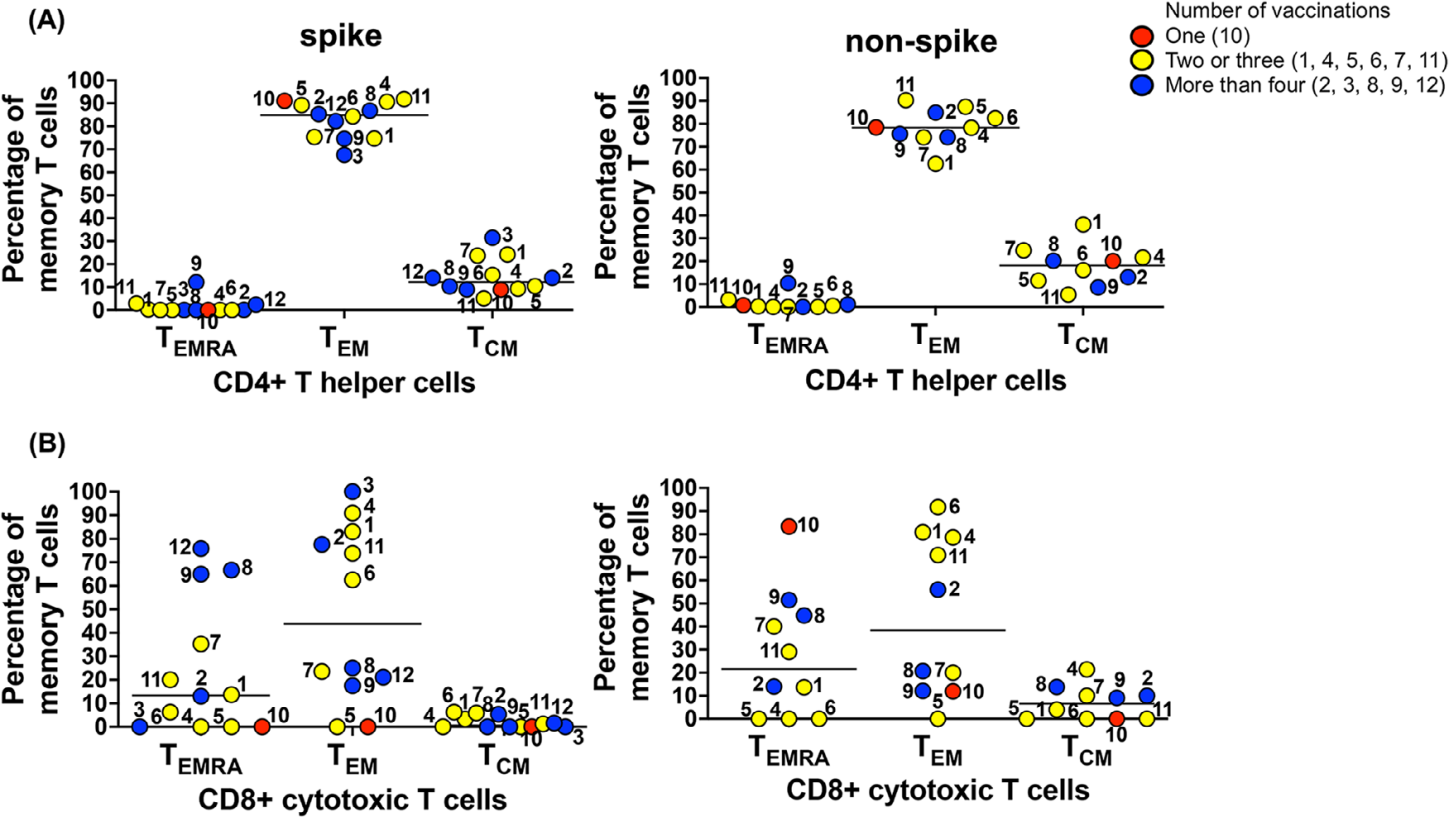
T cell memory phenotypes in RA subjects who received a different number of vaccine injections. (A) CD4^+^ Th memory responses to spike and nonspike peptide pools. Anti‐CD45RA and anti‐CCR7 were used to determine memory phenotypes on gated AIM^+^ CD4^+^ T helper cells. (B) CD8^+^ CTL memory responses. Each dot shows the percentage of memory populations: terminally differentiated effector T cells (CD45RA^+^ CCR7^−^ T_EMRA_), effector memory T cells (CD45RA^−^ CCR7^−^ T_EM_), and central memory T cells (CD45RA^−^ CCR7^+^ T_CM_). The number of vaccinations each subject received is denoted by the dot color. The solid line indicates the median percentage of memory T cells.

Terminally differentiated effector memory T cells (T_EMRA_), effector memory T cells (T_EM_), and central memory T cells (T_CM_) were analyzed on CD4^+^ and CD8^+^ AIM^+^ spike‐specific and nonspike‐specific T cells (Figure 2). We elected to study the T_EMRA_ population in more detail because of its significance in SARS‐CoV‐2‐specific T cell repertoire in COVID‐19 convalescent subjects and healthy vaccine recipients [7, 8, 9]. Within the CD4^+^ Th spike‐specific population (Figure 2A, left), T_EM_ cells were the largest spike‐specific T cell memory population observed among the subjects (median % 84.8). A lower level of CD4^+^ T_CM_ cells was also observed among the 12 subjects (median % 12.2). In contrast, the CD4^+^ T_EMRA_ memory cells were barely detected, and most subjects were negative (median % 0). The number of vaccine boosts did not correlate with the extent of the spike‐specific CD4^+^ T cell memory development.

When memory CD4^+^ T cell populations induced by nonspike peptide antigens were analyzed, an identical pattern of responses was observed (Figure 2A, right). Again, CD4+ T_EM_ cells were the largest T cell memory population induced against nonspike peptides (median % 78.4), while the CD4^+^ T_CM_ cells were again seen at a lower overall percentage (median % 18.1). CD4+ T_EMRA_ memory cells were barely detected among the subjects (median % 0.4).

Analysis of the memory phenotype of CD8^+^ spike‐specific CTL revealed some differences compared with CD4^+^ Th memory cells. As with CD4^+^ Th cells, T_EM_ CD8^+^ cells were observed among most of the subjects; however, the levels varied (Figure 2B, left). Six subjects expressed high percentages of CD8^+^ T_EM_ cells (>60%) while four subjects expressed intermediate levels (20%–30%). CD8^+^ T_EM_ cells could not be detected in two subjects. The overall median percentage of spike‐specific T_EM_ CD8^+^ cells among all subjects was 43.8.

Another interesting difference was observed in the CD8^+^ T_EMRA_ population. Although this memory population was virtually undetectable among the CD4^+^ T cells, spike‐specific CD8^+^ T_EMRA_ cells were detected in several subjects (Figure 2B, left). Three subjects expressed high percentages of CD8^+^ T_EMRA_ cells (>60%), four subjects had intermediate levels (10%–40%), and five subjects had low or undetectable levels. The overall median percentage of CD8+ T_EMRA_ cells in the 12 subjects was 13.4. The CD8+ T_CM_ cell response to spike peptide antigens was detected at very low levels in a few subjects, with most showing the absence of this memory cell population (median % 0) (Figure 2B, left). This finding differs from data previously reported in healthy vaccinated controls by our group and our collaborators [11, 12].

The pattern of CD8^+^ T cell memory responses to nonspike peptide antigens was similar to that observed against spike antigens (Figure 2B, right). CD8^+^ T_EM_ and CD8^+^ T_EMRA_ responses were observed in most subjects, with the median percentages measured at 38.4 and 21.5, respectively. Nonspike responses by CD8^+^ T_CM_ were again detected at lower levels (median % 6.5).

### 3.3 Treg Recognize SARS‐CoV‐2 Spike and Nonspike Peptide Epitopes in RA Subjects

The enumeration and characterization of spike‐ and nonspike‐specific Treg cells in RA vaccine recipients is relevant as a secondary endpoint to assess the potency of mRNA vaccination. In vaccinated healthy donors, our work showed that spike‐specific Treg represented the largest T‐cell population in circulation [12], data consistent with other studies [13, 14].

We analyzed SARS‐CoV‐2‐specific Treg by flow cytometry in PBMC cultures stimulated for 48 h with peptides. A longer stimulation was chosen to allow the expansion of Treg, which divide more slowly than CD4^+^ Th cells. CD4^+^ CD25^high^ Treg cell expansion after stimulation with spike and nonspike peptide pools was assessed with an SI (percentage of CD4^+^ CD25^high^ in stimulated cultures divided by the percentage CD4^+^ CD25^high^ in the unstimulated control).

**FIGURE 3:**
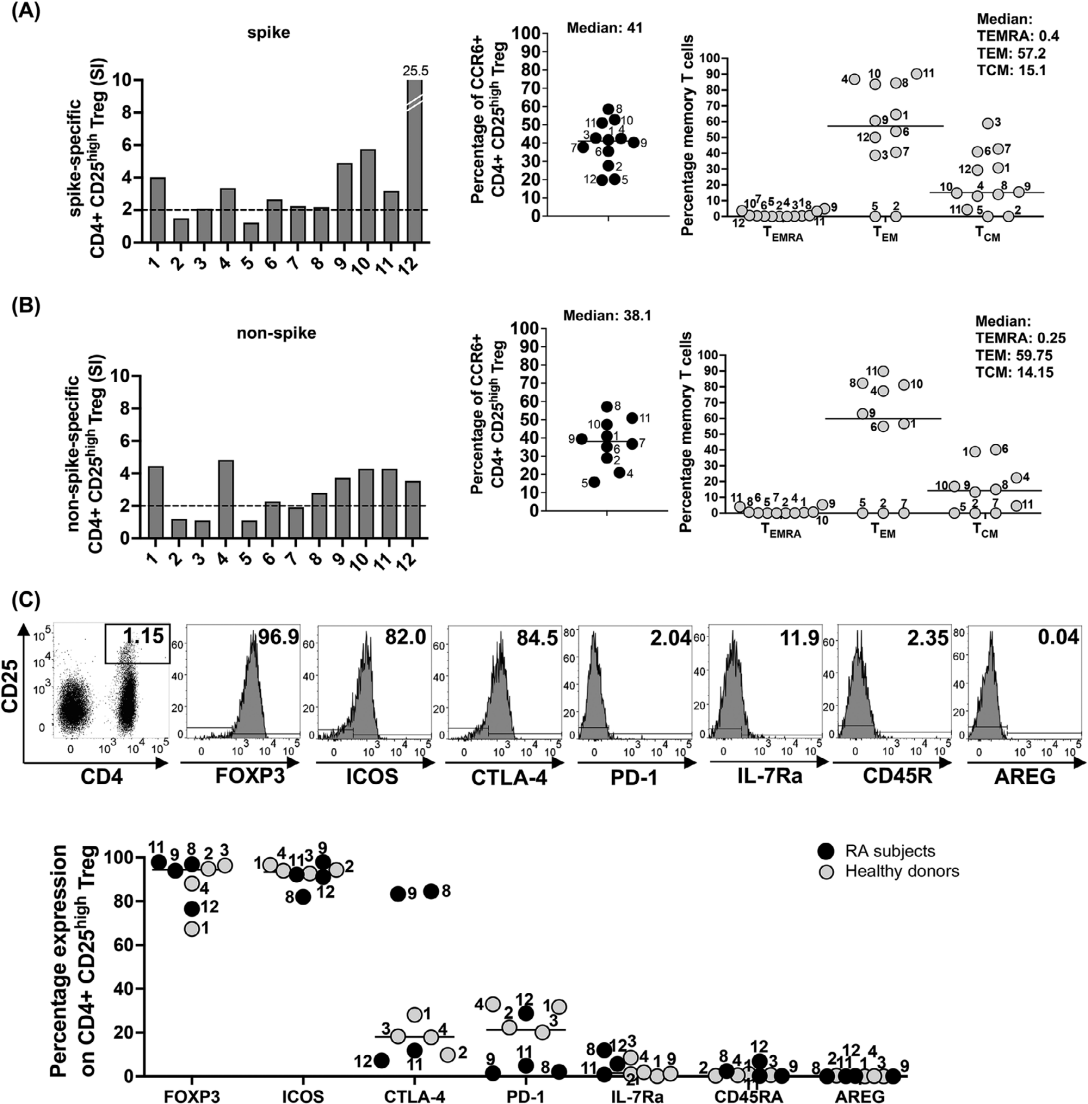
Characterization of spike and nonspike‐specific Treg. Treg were defined as CD4+ CD25^high^. In some subjects, Treg were additionally studied with FOXP3 intracellular, in combination with surface ICOS, CTLA‐4, and PD‐1. IL7R and CD45RA, expressed on pTreg, were also evaluated, and AREG. (A) Bar graphs on the left represent the percentage of CD3^+^ T cells, CD4^+^ CD25^high^ Treg in spike‐stimulated PBMC cultures. Data are presented as SI, marked on the graph bars as a dotted line. The middle panel shows the expression of CCR6 on Treg; the right panel shows the Treg memory. (B) Bar graphs on the left represent the percentage in PBMC of CD3^+^ T cells, CD4^+^ CD25^high^ Treg in nonspike‐stimulated PBMC, and data are presented as SI. The middle panel shows the expression of CCR6, and the right panel shows the Treg memory. (C) SARS‐CoV‐2 spike‐specific Treg were phenotypically characterized 48 h after cultures with spike peptides in PBMC from four RA subjects, namely 8, 9, 11, and 12 (filled symbols). Upper panel: representative flow images, Treg from RA subject 8. Four healthy donors served as controls (empty symbols). The markers were chosen to determine Treg ontogeny, possibly reverting from proinflammatory T cells (pTreg), or arising from naïve T cells primed in the lymph nodes. The percentage of Treg expressing ICOS, CTLA‐4, PD‐1, IL‐7R, CD45RA, and AREG is shown.

Ten of twelve subjects, namely 1, 3, 4, 6, 7, 8, 9, 10, 11, and 12, had spike‐specific Treg in circulation (Figure 3A, left graph). The CD4^+^ CD25^high^ Treg were CCR6^+^ (median % 41, Figure 3A, middle graph). Analysis of the T cell memory populations within the Treg cells suggested that spike‐specific T_EMRA_ cells were absent (median % 0.4, Figure 3A, right graph). T_EMRA_ were tested to capture Treg reverting from proinflammatory phenotypes. Higher percentages of T_EM_ Treg were observed (median % 57.2), as well as T_CM_ Treg (median % 15.1) (Figure 3A, right graph). Of interest in this analysis is subject 2, who developed RA after COVID‐19 infection. This individual did not have detectable levels of all three Treg memory populations.

Nonspike‐specific Treg cell expansion was observed in many subjects (Figure 3B, left graph). CCR6 was also expressed on nonspike‐specific Treg (median % 38.1), suggesting a similar tissue homing capacity as for the spike‐specific Treg (Figure 3B, middle graph). Memory T cells in the nonspike‐specific Treg population were also detected. T_EM_ Treg were numerous in all subjects except subjects 2, 5, and 7 (median % 59.7, Figure 3B, right graph). Nonspike‐specific T_CM_ Treg were detectable in subjects 1, 4, 6, 8, 9, 10, and 11, but not in subjects 2, 5, 7 (median % 14.2). T_EMRA_ cells were undetectable in most subjects (median % 0).

Next, we asked about the phenotype of the Treg cells by gating on intracellular FOXP3^+^ cells, then measuring cell surface expression of CTLA‐4, PD‐1, and ICOS. Other markers tested were CD45RA and IL‐7R, which defined peripherally‐induced Treg (pTreg) [15, 16, 17, 18]. Also of interest was the expression of amphiregulin (AREG) due to its potential role in Treg cell trafficking in the joints. AREG expression in Treg cells of acute COVID‐19 subjects inversely correlated with the severity of the disease [19]. AREG is a multifunctional molecule with a role in tissue repair by Treg [20], but is also involved with fibrosis [21]. In RA, AREG promotes the invasion of fibroblast‐like synoviocytes in the joints, exacerbating the disease [22].

For this study, PBMC cultures from RA subjects 8, 9, 11, and 12 (Table 1) were stimulated with SARS‐CoV‐2 spike peptides for 48 h before harvesting the cell preparation for flow cytometry. The choice of the subjects was unbiased, depending on PBMC numbers available after the AIM study. Four healthy donors, females, Caucasian, aged 26–55, served as controls in these experiments.

The results suggested that the phenotype of spike‐specific Treg FOXP3^+^ was slightly different in RA subjects versus healthy controls based on PD‐1 expression (Figure 3C). PD‐1 was undetectable on Treg from three of four RA subjects, namely 8, 9, and 11, in contrast with healthy controls (median % 3.5 in RA subjects versus median % 27 in healthy donors). ICOS was expressed on Treg in both cohorts (median % 91.7 in RA versus median % 94.1 in healthy donors). CTLA‐4 was expressed on Treg from both cohorts, but higher in the RA group due to high expression in two subjects (median % 47.6 in RA versus median % 18.1 in healthy donors). These subjects had measurable serum levels of anti‐citrullin (anti‐CCP) and anti‐nuclear (ANA) autoantibodies (Table 1). The markers that define peripherally induced Treg (pTreg), IL‐7R, and CD45RA, were negative (Figure 3C), supporting previous data from our group [12]. AREG was not expressed on the cell surface of spike‐specific Treg.

### 3.4 CD4^−^ CD8^−^ Double‐Negative T Cells Recognized SARS‐CoV‐2 Peptides and Differentiated into CD8^+^ Upon Stimulation

Analyzing CD4^+^ Th and CD8^+^ CTL responses, we realized that a large number of CD3^+^ T cells were CD4**^−^** CD8**^−^** DN. We then carefully enumerated activated CD4**^−^** CD8**^−^** DN T cells in the AIM assay.

**FIGURE 4:**
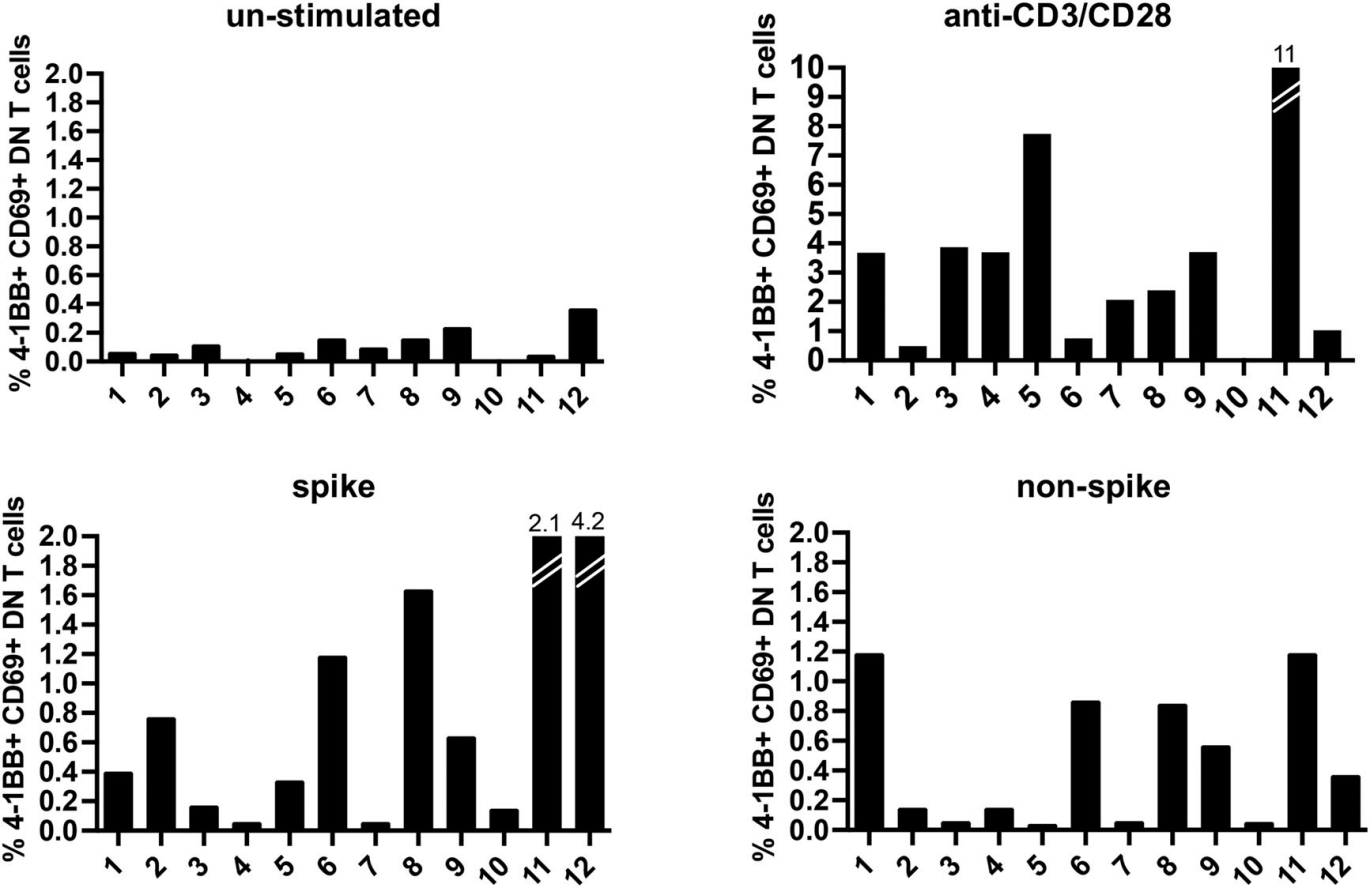
CD4^−^ CD8^−^ double negative (DN) T cells with a mature T cell receptor (TcR) developed after vaccination. Several CD3^+^ T cells were CD4^−^ CD8^−^ DN in PBMC from the twelve RA subjects studied for the recognition of the spike and nonspike peptide epitopes. Agonistic antibodies to CD3 and CD28 served as controls for CD3 signaling. The percentage of DN T cells in unstimulated PBMC, the percentage of DN T cells that responded to mitogenic stimulation with anti‐CD3/anti‐CD28, to the spike peptide epitopes, and to the nonspike peptide epitopes has been measured as the expression of 4‐1BB and CD69, markers that we used to enumerate CD8^+^ T cells.

DN expressed activation markers that we used to study CD8^+^ T cells, 4‐1BB, and CD69 (Figure 4). DN activation was evaluated in the unstimulated controls, in response to agonistic CD3 and CD28, and in response to spike and nonspike peptide epitopes (Figure 4).

The results are presented as percentages of CD4**^−^** CD8**^−^** DN T cells that responded to stimulation, rather than the S.I., because of the numerous AIM^+^ cells in the unstimulated control, which biased the read‐out (Figure 4).

A significant number of DN became activated in CD3/CD28‐stimulated PBMC cultures, and strongly expanded upon stimulation with spike and nonspike peptide epitopes (Figure 4).

To further investigate the specificity of DN T cells, their possible linear differentiation depending upon TcR signaling, we sorted by flow cytometry CD4**^−^** CD8**^−^** DN T cells from four additional RA subjects (subjects 13–16, Table 1), together with autologous, activated, CD11c^+^ CD11b^+^ CD86^+^ myeloid DC that served as antigen presenting cells (APC) in co‐cultures experiments. FACS sorting strategies are presented in Figure S1.

After sorting, DN T cells were plated in three experimental conditions: (a) stimulated with agonistic antibodies to CD3 and CD28, (b) co‐cultured with autologous myeloid DC in the absence of SARS‐CoV‐2 peptides, (c) co‐cultured with autologous DC, pulsed with spike peptide epitopes.

Twenty‐four hours later, supernatants were collected to measure IL‐2 secretion, before feeding with IL‐2. Seventy‐two hours after stimulation, cells were harvested to study by flow cytometry the activation stage, their phenotype, and a possible polarization toward a CD4^+^ or CD8^+^ single‐positive T cell.

**FIGURE 5:**
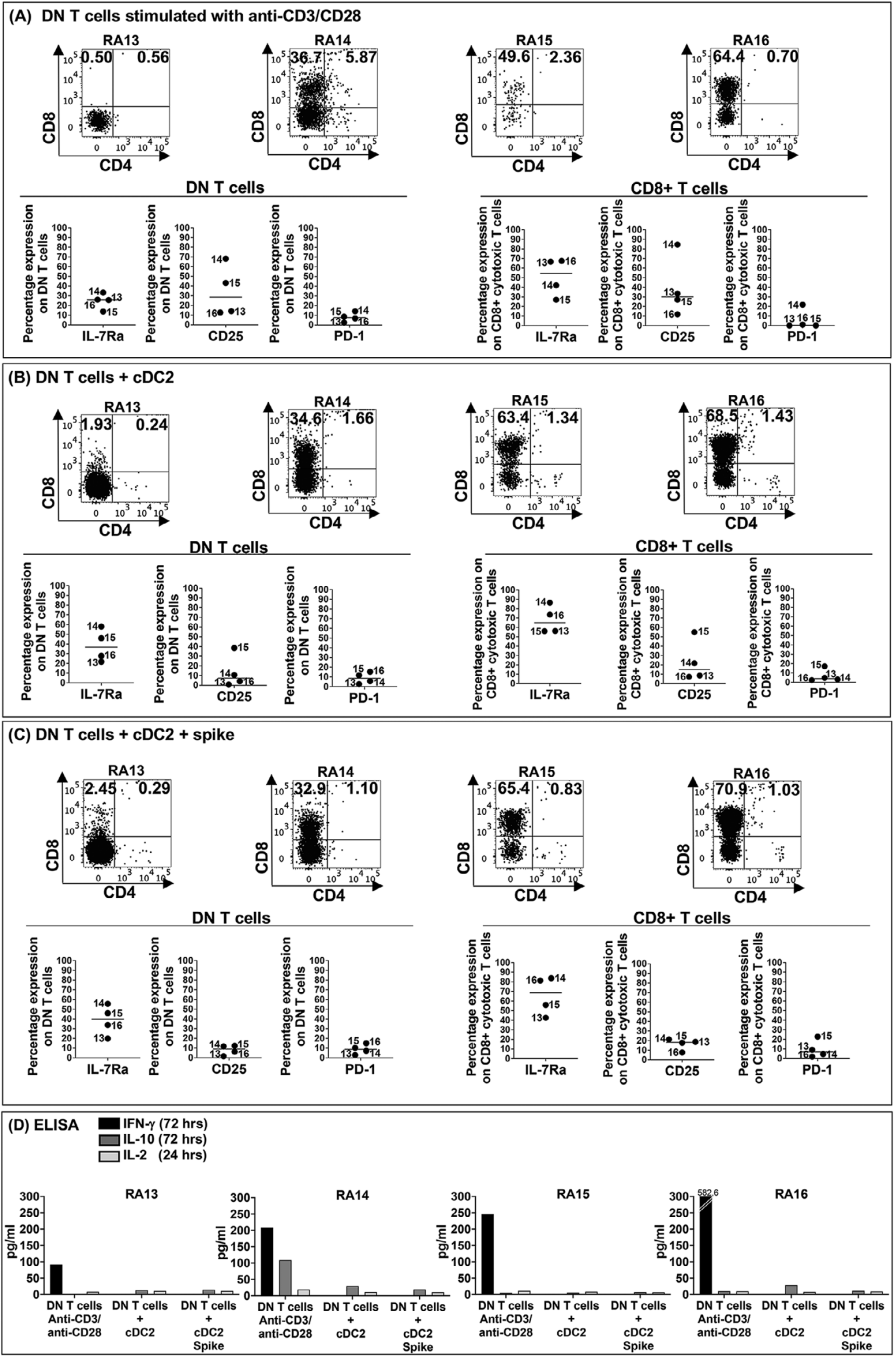
Phenotype and linear differentiation of CD4^−^ CD8^−^ DN T cells. PBMC obtained from four additional RA subjects, namely 13–16, were used to sort CD3^+^ CD4^−^ CD8‐DN T cells together with CD11c^+^ CD11b^+^ CD86^+^ activated myeloid DC for co‐cultures and in vitro stimulation. (A) DN T cells alone, stimulated with agonistic antibodies to CD3/CD28, were harvested 72 h after cultures for phenotypical characterization. Upper panels: polarization toward a CD8^+^ T cell phenotype. Lower panels: expression of IL‐7R, IL‐2R, and PD‐1 in DN T cells, and in single positive CD8^+^ T cells. (B) DN T cells co‐cultured with autologous cDC2: upper panels: polarization toward a CD8^+^ T cell phenotype. Lower panels: expression of IL‐7R, IL‐2R, and PD‐1. (C) DN T cells co‐cultured with autologous myeloid DC pulsed with spike peptide epitopes: upper panels: polarization toward a CD8^+^ T cell phenotype. Lower panels: expression of IL‐7R, IL‐2R, and PD‐1. (D) Measurement of lymphokines in culture supernatants by ELISA: IL‐2 was measured 24 h after stimulation, and IFNγ and IL‐10, 72 h after stimulation.

In four of four RA subjects, DN T cells became CD8^+^ T cells under CD3/CD28 stimulation, with some variability, but with a clear pattern of differentiation (Figure 5A). DN stimulated with CD3/CD28‐expressed IL7‐R, suggesting active division and expansion (Figure 5A). The IL‐2R CD25 was measurable in CD3/CD28 (Figure 5A). PD‐1, a marker previously described in DN T cells derived from exhausted CD8^+^ T cells in human autoimmune conditions [23, 24] and from exhausted autoreactive CD8^+^ T cells in murine models [25, 26], was not significantly expressed on DN, nor on CD8^+^ newly differentiated T cells (Figure 5A).

DN T cells differentiated into CD8^+^ T cells also when co‐cultured with autologous myeloid DC in the absence of spike peptide epitopes, and expressed IL‐7R, but low CD25 (Figure 5B), suggesting cell division and the recognition of endogenous antigens, but a lesser TcR engagement, compared with the direct stimulation of the CD3 complex.

Comparable results were obtained when DN were co‐cultured with myeloid DC pulsed with spike peptide epitopes, with expression of the IL‐7R and low CD25, again suggesting the need for a stronger TcR stimulus for a productive activation/expansion. Of interest, PD‐1 was poorly expressed on DN and newly differentiated CD8^+^ T cells in co‐cultures (Figure 5B,C).

The results suggested that DN T cells in these RA subjects did not arise from exhausted CD8^+^ T cells, but rather mobilized from the thymus with a mature TcR, and can respond to antigens.

When we looked at lymphokine secretion, none of the DN culture conditions showed IL‐2 production 24 h after stimulation (Figure 5D). However, a high amount of IFNγ was detectable in FACS‐sorted DN cultures stimulated with agonistic CD3/CD28 72 h after cultures, but not in DN/myeloid DC co‐cultures, with or without antigens.

The lymphokine profile suggested that the strength of the TcR signaling on DN T cells should be sufficient to lead to IFNγ secretion and proinflammatory functions. IL‐10 was undetectable in all the experimental conditions (Figure 5D).

## 4 Discussion

The efficacy of mRNA‐based vaccines for COVID‐19 protection in autoimmune patients under immunosuppressive medication has been debated, with spurious data reporting low antibody responses [27, 28].

The potential role of SARS‐CoV‐2 infection and/or vaccination in exacerbating or inducing autoimmunity is still under investigation [5, 6].

Here, we studied the SARS‐CoV‐2‐specific T cell responses in RA, a classical model of systemic autoimmunity. RA subjects in this study received different doses of mRNA‐based vaccine (from 1 to 7), were of different ages, ethnicity, and affected by other autoimmune co‐morbidities. The therapeutic schedules were very diversified. None of the RA subjects in this cohort experienced clinical relapses after vaccination.

All the subjects developed spike‐specific CD4^+^ Th responses. The difference in the magnitude of the CD4^+^ Th expansion was not correlated with the number of injections, but rather with the HLA alleles of the subjects that potentially bind immunodominant peptides with a lesser affinity.

Ten of twelve subjects induced CD8^+^ T cell responses to the spike peptide epitopes.

Five subjects of twelve in the cohort did not report COVID‐19 infection before the study. However, T cell responses to nonspike peptide epitopes suggested asymptomatic COVID‐19 infection in three subjects. T cell responses to the nonspike regions of the SARS‐CoV‐2 virus are a reliable method to address COVID‐19 exposure, rather than measuring antibody responses [10]. It is important to note that pre‐existing cross‐reactive memory T cells from prior coronavirus exposures, which were not systematically assessed in our study, could also influence the magnitude and quality of responses observed.

Nonspike‐specific CD8^+^ T cells were less numerous than CD4^+^ Th, in subjects exposed to the SARS‐CoV‐2 virus.

The expression of CCR6, a chemokine receptor that allows T cell homing to the endothelial side of vessels, lungs, gut, myometrium, and vagina, was expressed on CD4^+^ Th cells and Treg, but also on CD8^+^ T cells, differing from our previous study in healthy vaccinated controls [12]. CCR6^+^ CD8^+^ SARS‐CoV‐2‐specific T cells can potentially infiltrate tissues and worsen the inflammation in RA when re‐exposed to COVID‐19 infection. No difference with this homing marker was observed between spike and nonspike‐specific T cells, CD4^+^ Th or CD8^+^ CTL.

When we looked at T cell memory, we found a good CD4^+^ Th memory development to the spike and nonspike peptide epitopes: CD4^+^ T_EMRA_ low, and CD4^+^ T_EM_ and T_CM_ numerous.

The CD8^+^ memory to the spike and nonspike peptide epitopes suggested that CD8^+^ T_EMRA_, T_EM,_ and T_CM_ expanded in most subjects, with some differences. CD8+ T_EM_ and T_CM_ in RA were higher than the CD8^+^ T cell memory reported in healthy vaccine recipients and COVID‐19 convalescent adults [11], convalescent pregnant women [29], and in children with multisystemic inflammatory syndrome (MIS‐C) [30]. In all these human cohorts, CD8^+^ T cell memory was confined to the T_EMRA_ lineage. However, further characterization is required to understand the contributing factors that may influence T cell memory development, such as tissue factors, peptide binding affinity to the TCR, and IL‐15 receptor expression [31].

In acute COVID‐19 infection, Treg were a mechanism to mitigate the severity of the symptoms [32]. In support of these findings, in RA, subjects who had asymptomatic COVID‐19 infection showed numerous nonspike‐specific Treg in circulation, as for subjects who had symptomatic COVID‐19. Of interest, one of the two subjects in the cohort that did not expand a spike‐specific Treg response developed RA after COVID‐19, stressing the importance of spike‐specific Treg, not only in the context of SARS‐CoV‐2 protection, but, at large, in controlling immune tolerance.

SARS‐CoV‐specific Treg expanded in vaccinated RA subjects, as previously reported by our group in healthy vaccine recipients [12]. Spike and nonspike‐specific Treg did not express the IL‐7R and CD45RA, as peripherally‐induced Treg (pTreg). The ontogeny of spike‐specific Treg is currently under investigation in our, and other laboratories. These cells are unlikely to revert from Th17 or other proinflammatory T cells repeatedly stimulated, but are rather primed in secondary lymphoid organs. SARS‐CoV‐2‐specific Treg phenotypically appeared as natural Treg (nTreg).

The IL‐2 requirements and the expression of PD‐1 by SARS‐CoV‐2‐specific Treg were different in RA compared with healthy vaccinated controls, although we acknowledge that, because of the low number of samples available for the assay, the results should be interpreted with caution. However, our data are in agreement with the idea that human CD4+ IL‐10‐producing T cells are heterogeneous [33]. In fact, Treg adapt to immune metabolic checkpoints, and are deeply influenced by the homing and the microbiome in the environment [34]. Treg plasticity is so extensive that, in a skin murine model, Treg cells reverted to follicular Th cells [35].

Spike‐specific Treg from RA subjects were low in the expression of PD‐1 in most subjects, suggesting an inefficient crosstalk with proinflammatory innate immune cells and T cells [36]. We acknowledge that the surface expression of AREG, downstream of NOTCH‐4, was compromised in acutely infected COVID‐19 adults, where the lack of AREG expression correlated with severe symptomatology [19]. In RA, this marker on T cells may lead to different, contrasting outcomes. Although relevant in tissue repair [20], AREG can promote fibrosis in the joints [21] and the invasion of fibroblast‐like synoviocytes in the joints, exacerbating the disease [22].

We found that AREG is not expressed on SARS‐CoV‐2‐specific Treg, indicating differences with the Treg described in the acute phase of COVID‐19 [19]. In our study, spike‐ and nonspike‐specific Treg may not reach the synovial site, but, since they express CCR6, they could home to a variety of other tissues.

A unique feature of the SARS‐CoV‐2 T cell response in RA vaccine recipients was the expansion of numerous spike and nonspike‐specific CD4^−^ CD8^−^ DN T cells.

In autoimmunity, TCRαβ^+^ CD3^+^ CD4^−^ CD8^−^ T cells were described as a small subset of peripheral T cells, capable of expanding in several autoimmune conditions, such as psoriasis, Sjogren syndrome, systemic lupus erythematosus (SLE) (reviewed in [37]).

The contribution of DN T cells to the pathogenesis of autoimmune diseases depended upon a proinflammatory phenotype, and the secretion of proinflammatory lymphokines, like IL‐1 β, IL‐17, and IFNγ, and possibly autoreactive TcRs that strongly bind amino acid residues on HLA molecules.

In murine models, the ontogeny of DN T cells in healthy mice depended upon the exhaustion of CD8^+^ cytotoxic T cells, which downregulated the CD8 co‐receptor and expressed PD‐1 [25, 26].

We studied DN T cells in this RA and found a much higher percentage of CD3^+^ CD4^−^ CD8^−^ DN T cells in the unstimulated control than in cohorts previously studied [30, 38]. DN strongly responded to agonistic CD3/CD28 antibodies, and to spike and nonspike peptide epitopes, suggesting TcR maturity and recognition of a large range of specificities.

When FACS‐sorted, DN T cells did not secrete IL‐2, either when stimulated with CD3/CD28, nor when co‐cultured with activated autologous myeloid cDC2, with or without antigens. After IL‐2 feeding, a considerable percentage of DN T cells polarized toward single positive CD8^+^ T cells, with some variability from subject to subject, under all the stimulatory conditions. These results suggested that the IL‐2 secretion by other activated T cells supported the viability and expansion of DN T cells in RA subjects. Further phenotypical analysis revealed that PD‐1 was not expressed on DN, nor CD8^+^ single‐positive T cells, but the IL‐7R was very high, suggesting ongoing activation and expansion. Of interest, when we measured IFNγ and IL‐10 in culture supernatants, we found that only CD3/CD28‐stimulated CD4^−^ CD8^−^ DN cultures, as well as newly differentiated CD8+ T cells, secreted IFNγ. No lymphokines were measured in co‐cultures with autologous cDC2, with or without antigens. Therefore, we believe that these T cells are not autoreactive in nature, but are capable of expanding and differentiating in the lymph nodes and secondary lymphoid organs when optimally stimulated by antigens, maybe in the context of overstimulation. We propose here a model where the strength of TcR occupancy should be high for DN T cells to become proinflammatory, possibly regulated in vivo by the antigen dose, HLA restriction, and repeated stimulations.

The enumeration of DN T cells in the SARS‐CoV‐2 model was not unique in RA. We reported DN T cells in MIS‐C, weeks after COVID‐19, and in pregnant women vaccinated multiple times for COVID‐19 protection that also received at the same time other vaccine formulations (Tetanus, Diphtheria, Pertussis, respiratory syncytial virus [RSV], and Flu) [30, 38]. DN T cells, becoming CD8^+^ cells with a proinflammatory phenotype, may cause undesirable side effects of vaccination in initiating or exacerbating autoimmunity.

We recognize the limitations of this work, mainly because of the lack of access to lymphoid organs and tissue samples. Also, the lack of information on the timing of the natural COVID‐19 infection, particularly asymptomatic infections, may have impacted T cell memory.

A strength of the paper is the deeper characterization of the spike and nonspike‐specific Treg and their unique phenotype in SARS‐CoV‐2 RA vaccine recipients, as well as the description of a CD4^−^CD8^−^ DN T cell population that differentiated into CD8^+^ T cells in the periphery.

Interestingly, DN T cells, fully functional and capable of upregulating CD8^+^ T cell activation markers, appear to be consistently detected in unique human cohorts, namely very inflamed pediatric patients, pregnant women, and now autoimmune subjects.

## Author Contributions

Jaeyoon Song performed the experiment under Alessandra Franco's supervision, Monica Guma enrolled the RA subjects, Merhnaz Agili Seyede coordinated the sample collection from the Rheumatology clinic, Ricardo da Silva Antunes and Alessandro Sette designed and provided spike and nonspike SARS‐CoV‐2 peptide epitopes, Alessandra Franco designed the experiments, provided funds for the projects, supervised the execution of the experiments, and wrote the paper.

## Funding

Praespero, an Autoimmune Research Fund to AF, by the Gordon and Marylin Macklin Foundation to AF, by the NIH NIAID 75N9301900065 to AS.

## Ethics Statement

The study has received ethical approval from the institutional review board. It also confirms that informed consent was obtained from participants.

## Institutional Review Board Statement

The study was conducted according to the guidelines of the Declaration of Helsinki and approved by the Institutional Review Board (or Ethics Committee) of the University of California San Diego.

## Informed Consent Statement

Informed consent was obtained from all subjects involved in the study. Written informed consent has been obtained from the subjects to obtain blood samples.

## Conflicts of Interest

The authors declare no conflicts of interest.

## Supporting information




**Supporting File**: eji70105‐sup‐0001‐SuppMat.pdf.

## Data Availability

The authors confirm that the data supporting the findings of this study are available within the article.
