# Supplementary material for: Characterization of the SARS‐CoV‐2‐Specific T Cell Responses in Rheumatoid Arthritis Subjects Vaccinated for COVID‐19 Protection
Source: Eur J Immunol. 2025 Dec 19;55(12):e70105. doi: 10.1002/eji.70105 (PMC12716210; doi:10.1002/eji.70105)
Supplement: Supplementary file 1 — Supporting File: eji70105‐sup‐0001‐SuppMat.pdf. [file EJI-55-e70105-s001.pdf]

Supplementary Figure 1. Gating strategies to FACS-sort CD4-CD8- DN T cells and CD11c+CD11b+ myeloid DC

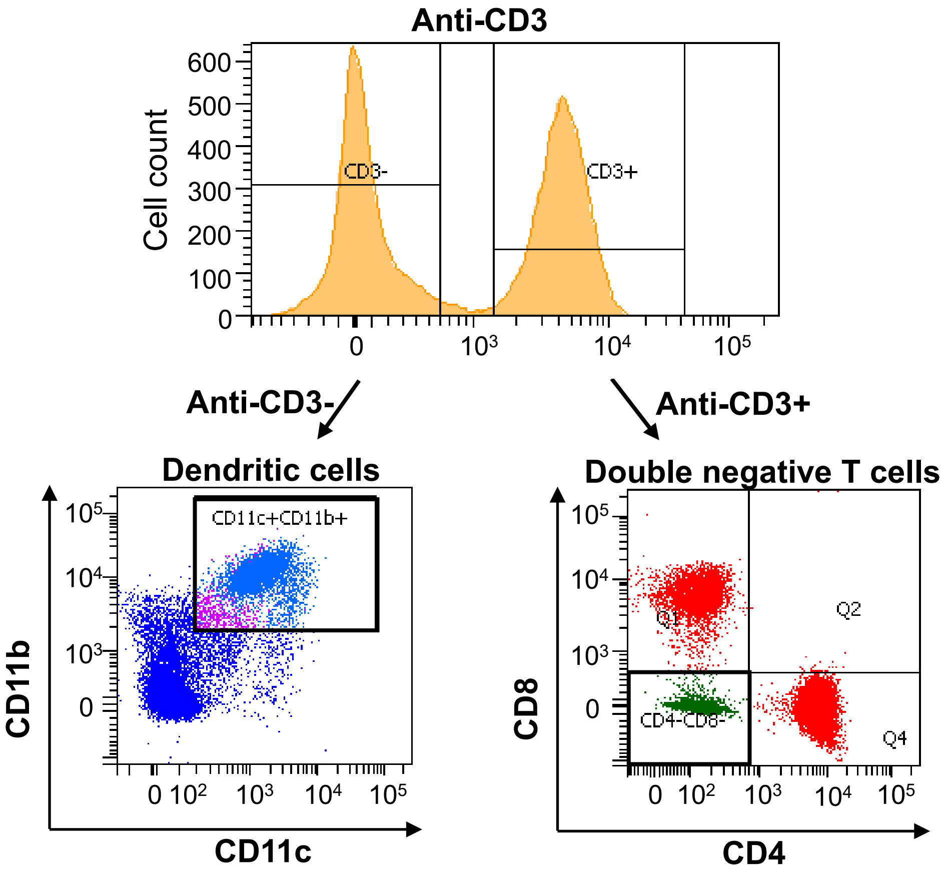

To establish autologous myeloid dendritic cells and CD4-CD8- DN T cell co-cultures, PBMC were separated by Ficoll-Hypaque, to characterize cell populations with specific monoclonal antibodies. The first gate defined CD3+ T cells (upper panel). Myeloid DC were obtained from the CD3- cell populations, gating on double positive CD11c+ CD11b+ cells (left lower panels). Single positive CD4+ and CD8+ T cells were excluded from CD3+ T cells to FACS-sort CD4- CD8- DN T cells (right lower panel).

Supplementary Table 1. Spike peptides

Peptide	Description	Len	Antigen	Start	End
1	MFVFLVLLPLVSSQC	15	S	1	15
2	VLLPLVSSQCVNLTT	15	S	6	20
3	VSSQCVNLTTRTQLP	15	S	11	25
4	VNLTTRTQLPPAYTN	15	S	16	30
5	RTQLPPAYTNSFTRG	15	S	21	35
6	PAYTNSFTRGVYYPD	15	S	26	40
7	SFTRGVYYPDKVFRS	15	S	31	45
8	VYYPDKVFRSSVLHS	15	S	36	50
9	KVFRSSVLHSTQDLF	15	S	41	55
10	SVLHSTQDLFLPFFS	15	S	46	60
11	TQDLFLPFFSNVTWF	15	S	51	65
12	LPFFSNVTWFHAIHV	15	S	56	70
13	NVTWFHAIHVSGTNG	15	S	61	75
14	HAIHVSGTNGTKRFD	15	S	66	80
15	SGTNGTKRFDNPVLP	15	S	71	85
16	TKRFDNPVLPFNDGV	15	S	76	90
17	NPVLPFNDGVYFAST	15	S	81	95
18	FNDGVYFASTEKSNI	15	S	86	100
19	YFASTEKSNIIRGWI	15	S	91	105
20	EKSNIIRGWIFGTTL	15	S	96	110
21	IRGWIFGTTLDSKTQ	15	S	101	115
22	FGTTLDSKTQSLLIV	15	S	106	120
23	DSKTQSLLIVNNATN	15	S	111	125
24	SLLIVNNATNVVIKV	15	S	116	130
25	NNATNVVIKVCEFQF	15	S	121	135
26	VVIKVCEFQFCNDPF	15	S	126	140
27	CEFQFCNDPFLGVYY	15	S	131	145
28	CNDPFLGVYYHKNNK	15	S	136	150
29	LGVYYHKNNKSWMES	15	S	141	155
30	HKNNKSWMESEFRVY	15	S	146	160
31	SWMESEFRVYSSANN	15	S	151	165
32	EFRVYSSANNCTFEY	15	S	156	170
33	SSANNCTFEYVSQPF	15	S	161	175
34	CTFEYVSQPFLMDLE	15	S	166	180
35	VSQPFLMDLEGKQGN	15	S	171	185
36	LMDLEGKQGNFKNLR	15	S	176	190
37	GKQGNFKNLREFVFK	15	S	181	195
38	FKNLREFVFKNIDGY	15	S	186	200
39	EFVFKNIDGYFKIYS	15	S	191	205
40	NIDGYFKIYSKHTPI	15	S	196	210
41	FKIYSKHTPINLVRD	15	S	201	215
42	KHTPINLVRDLPQGF	15	S	206	220
43	NLVRDLPQGFSALEP	15	S	211	225
44	LPQGFSALEPLVDLP	15	S	216	230
45	SALEPLVDLPIGINI	15	S	221	235
46	LVDLPIGINITRFQT	15	S	226	240
47	IGINITRFQTLLALH	15	S	231	245
48	TRFQTLLALHRSYLT	15	S	236	250
49	LLALHRSYLTPGDSS	15	S	241	255
50	RSYLTPGDSSSGWTA	15	S	246	260
51	PGDSSSGWTAGAAAY	15	S	251	265
52	SGWTAGAAAYYVGYL	15	S	256	270
53	GAAAYYVGYLQPRTF	15	S	261	275
54	YVGYLQPRTFLLKYN	15	S	266	280
55	QPRTFLLKYNENGTI	15	S	271	285
56	LLKYNENGTITDAVD	15	S	276	290
57	ENGTITDAVDCALDP	15	S	281	295
58	TDAVDCALDPLSETK	15	S	286	300
59	CALDPLSETKCTLKS	15	S	291	305
60	LSETKCTLKSFTVEK	15	S	296	310
61	CTLKSFTVEKGIYQT	15	S	301	315
62	FTVEKGIYQTSNFRV	15	S	306	320
63	GIYQTSNFRVQPTES	15	S	311	325
64	SNFRVQPTESIVRFP	15	S	316	330
65	QPTESIVRFPNITNL	15	S	321	335
66	IVRFPNITNLCPFGE	15	S	326	340
67	NITNLCPFGEVFNAT	15	S	331	345
68	CPFGEVFNATRFASV	15	S	336	350
69	VFNATRFASVYAWNR	15	S	341	355
70	RFASVYAWNRKRISN	15	S	346	360
71	YAWNRKRISNCVADY	15	S	351	365
72	KRISNCVADYSVLYN	15	S	356	370
73	CVADYSVLYNSASFS	15	S	361	375
74	SVLYNSASFSTFKCY	15	S	366	380
75	SASFSTFKCYGVSPT	15	S	371	385
76	TFKCYGVSPTKLNDL	15	S	376	390
77	GVSPTKLNDLCFTNV	15	S	381	395
78	KLNDLCFTNVYADSF	15	S	386	400
79	CFTNVYADSFVIRGD	15	S	391	405
80	YADSFVIRGDEVRQI	15	S	396	410
81	VIRGDEVRQIAPGQT	15	S	401	415
82	EVRQIAPGQTGKIAD	15	S	406	420
83	APGQTGKIADYNYKL	15	S	411	425
84	GKIADYNYKLPDDFT	15	S	416	430
85	YNYKLPDDFTGCVIA	15	S	421	435
86	PDDFTGCVIAWNSNN	15	S	426	440
87	GCVIAWNSNNLDSKV	15	S	431	445
88	WNSNNLDSKVGGNYN	15	S	436	450
89	LDSKVGGNYNYLYRL	15	S	441	455
90	GGNYNYLYRLFRKSN	15	S	446	460
91	YLYRLFRKSNLKPFE	15	S	451	465
92	FRKSNLKPFERDIST	15	S	456	470
93	LKPFERDISTEIYQA	15	S	461	475
94	RDISTEIYQAGSTPC	15	S	466	480
95	EIYQAGSTPCNGVEG	15	S	471	485
96	GSTPCNGVEGFNCYF	15	S	476	490
97	NGVEGFNCYFPLQSY	15	S	481	495
98	FNCYFPLQSYGFQPT	15	S	486	500
99	PLQSYGFQPTNGVGY	15	S	491	505
100	GFQPTNGVGYQPYRV	15	S	496	510
101	NGVGYQPYRVVVLSF	15	S	501	515
102	QPYRVVVLSFELLHA	15	S	506	520
103	VVLSFELLHAPATVC	15	S	511	525
104	ELLHAPATVCGPKKS	15	S	516	530
105	PATVCGPKKSTNLVK	15	S	521	535
106	GPKKSTNLVKNKCVN	15	S	526	540
107	TNLVKNKCVNFNFNG	15	S	531	545
108	NKCVNFNFNGLTGTG	15	S	536	550
109	FNFNGLTGTGVLTES	15	S	541	555
110	LTGTGVLTESNKKFL	15	S	546	560
111	VLTESNKKFLPFQQF	15	S	551	565
112	NKKFLPFQQFGRDIA	15	S	556	570
113	PFQQFGRDIADTTDA	15	S	561	575
114	GRDIADTTDAVRDPQ	15	S	566	580
115	DTTDAVRDPQTLEIL	15	S	571	585
116	VRDPQTLEILDITPC	15	S	576	590
117	TLEILDITPCSFGGV	15	S	581	595
118	DITPCSFGGVSVITP	15	S	586	600
119	SFGGVSVITPGTNTS	15	S	591	605
120	SVITPGTNTSNQVAV	15	S	596	610
121	GTNTSNQVAVLYQDV	15	S	601	615
122	NQVAVLYQDVNCTEV	15	S	606	620
123	LYQDVNCTEVPVAIH	15	S	611	625
124	NCTEVPVAIHADQLT	15	S	616	630
125	PVAIHADQLTPTWRV	15	S	621	635
126	ADQLTPTWRVYSTGS	15	S	626	640
127	PTWRVYSTGSNVFQT	15	S	631	645
128	YSTGSNVFQTRAGCL	15	S	636	650
129	NVFQTRAGCLIGAEH	15	S	641	655
130	RAGCLIGAEHVNNSY	15	S	646	660

Peptide	Description	Len	Antigen	Start	End
131	IGAEHVNNSYECDIP	15	S	651	665
132	VNNSYECDIPIGAGI	15	S	656	670
133	ECDIPIGAGICASYQ	15	S	661	675
134	IGAGICASYQTQTNS	15	S	666	680
135	CASYQTQTNSPRRAR	15	S	671	685
136	TQTNSPRRARSVASQ	15	S	676	690
137	PRRARSVASQSIIAY	15	S	681	695
138	SVASQSIIAYTMSLG	15	S	686	700
139	SIIAYTMSLGAENSV	15	S	691	705
140	TMSLGAENSVAYSNN	15	S	696	710
141	AENSVAYSNNSIAIP	15	S	701	715
142	AYSNNSIAIPTNFTI	15	S	706	720
143	SIAIPTNFTISVTTE	15	S	711	725
144	TNFTISVTTEILPVS	15	S	716	730
145	SVTTEILPVSMTKTS	15	S	721	735
146	ILPVSMTKTSVDCTM	15	S	726	740
147	MTKTSVDCTMYICGD	15	S	731	745
148	VDCTMYICGDSTECS	15	S	736	750
149	YICGDSTECSNLLLQ	15	S	741	755
150	STECSNLLLQYGSFC	15	S	746	760
151	NLLLQYGSFCTQLNR	15	S	751	765
152	YGSFCTQLNRALTGI	15	S	756	770
153	TQLNRALTGIAVEQD	15	S	761	775
154	ALTGIAVEQDKNTQE	15	S	766	780
155	AVEQDKNTQEVFAQV	15	S	771	785
156	KNTQEVFAQVKQIYK	15	S	776	790
157	VFAQVKQIYKTPPIK	15	S	781	795
158	KQIYKTPPIKDFGGF	15	S	786	800
159	TPPIKDFGGFNFSQI	15	S	791	805
160	DFGGFNFSQILPDPS	15	S	796	810
161	NFSQILPDPSKPSKR	15	S	801	815
162	LPDPSKPSKRSFIED	15	S	806	820
163	KPSKRSFIEDLLFNK	15	S	811	825
164	SFIEDLLFNKVTLAD	15	S	816	830
165	LLFNKVTLADAGFIK	15	S	821	835
166	VTLADAGFIKQYGDC	15	S	826	840
167	AGFIKQYGDCLGDIA	15	S	831	845
168	QYGDCLGDIAARDLI	15	S	836	850
169	LGDIAARDLICAQKF	15	S	841	855
170	ARDLICAQKFNGLTV	15	S	846	860
171	CAQKFNGLTVLPPLL	15	S	851	865
172	NGLTVLPPLLTDEMI	15	S	856	870
173	LPPLLTDEMIAQYTS	15	S	861	875
174	TDEMIAQYTSALLAG	15	S	866	880
175	AQYTSALLAGTITSG	15	S	871	885
176	ALLAGTITSGWTFGA	15	S	876	890
177	TITSGWTFGAGAALQ	15	S	881	895
178	WTFGAGAALQIPFAM	15	S	886	900
179	GAALQIPFAMQMAYR	15	S	891	905
180	IPFAMQMAYRFNGIG	15	S	896	910
181	QMAYRFNGIGVTQNV	15	S	901	915
182	FNGIGVTQNVLYENQ	15	S	906	920
183	VTQNVLYENQKLIAN	15	S	911	925
184	LYENQKLIANQFNSA	15	S	916	930
185	KLIANQFNSAIGKIQ	15	S	921	935
186	QFNSAIGKIQDSLSS	15	S	926	940
187	IGKIQDSLSSTASAL	15	S	931	945
188	DSLSSTASALGKLQD	15	S	936	950
189	TASALGKLQDVVNQN	15	S	941	955
190	GKLQDVVNQNAQALN	15	S	946	960
191	VVNQNAQALNTLVKQ	15	S	951	965
192	AQALNTLVKQLSSNF	15	S	956	970
193	TLVKQLSSNFGAISS	15	S	961	975
194	LSSNFGAISSVLNDI	15	S	966	980
195	GAISSVLNDILSRLD	15	S	971	985

Peptide	Description	Len	Antigen	Start	End
196	VLNDILSRLDKVEAE	15	S	976	990
197	LSRLDKVEAEVQIDR	15	S	981	995
198	KVEAEVQIDRLITGR	15	S	986	1000
199	VQIDRLITGRLQSLQ	15	S	991	1005
200	LITGRLQSLQTYVTQ	15	S	996	1010
201	LQSLQTYVTQQLIRA	15	S	1001	1015
202	TYVTQQLIRAAEIRA	15	S	1006	1020
203	QLIRAAEIRASANLA	15	S	1011	1025
204	AEIRASANLAATKMS	15	S	1016	1030
205	SANLAATKMSECVLG	15	S	1021	1035
206	ATKMSECVLGQSKRV	15	S	1026	1040
207	ECVLGQSKRVDFCGK	15	S	1031	1045
208	QSKRVDFCGKGYHLM	15	S	1036	1050
209	DFCGKGYHLMSFPQS	15	S	1041	1055
210	GYHLMSFPQSAPHGV	15	S	1046	1060
211	SFPQSAPHGVVFLHV	15	S	1051	1065
212	APHGVVFLHVTYVPA	15	S	1056	1070
213	VFLHVTYVPAQEKNF	15	S	1061	1075
214	TYVPAQEKNFTTAPA	15	S	1066	1080
215	QEKNFTTAPAICHDG	15	S	1071	1085
216	TTAPAICHDGKAHFP	15	S	1076	1090
217	ICHDGKAHFPREGVF	15	S	1081	1095
218	KAHFPREGVFVSNGT	15	S	1086	1100
219	REGVFVSNGTHWFVT	15	S	1091	1105
220	VSNGTHWFVTQRNFY	15	S	1096	1110
221	HWFVTQRNFYEPQII	15	S	1101	1115
222	QRNFYEPQIITTDNT	15	S	1106	1120
223	EPQIITTDNTFVSGN	15	S	1111	1125
224	TTDNTFVSGNCDVVI	15	S	1116	1130
225	FVSGNCDVVIGIVNN	15	S	1121	1135
226	CDVVIGIVNNTVYDP	15	S	1126	1140
227	GIVNNTVYDPLQPEL	15	S	1131	1145
228	TVYDPLQPELDSFKE	15	S	1136	1150
229	LQPELDSFKEELDKY	15	S	1141	1155
230	DSFKEELDKYFKNHT	15	S	1146	1160
231	ELDKYFKNHTSPDVD	15	S	1151	1165
232	FKNHTSPDVDLGDIS	15	S	1156	1170
233	SPDVDLGDISGINAS	15	S	1161	1175
234	LGDISGINASVVNIQ	15	S	1166	1180
235	GINASVVNIQKEIDR	15	S	1171	1185
236	VVNIQKEIDRLNEVA	15	S	1176	1190
237	KEIDRLNEVAKNLNE	15	S	1181	1195
238	LNEVAKNLNESLIDL	15	S	1186	1200
239	KNLNESLIDLQELGK	15	S	1191	1205
240	SLIDLQELGKYEQYI	15	S	1196	1210
241	QELGKYEQYIKWPWY	15	S	1201	1215
242	YEQYIKWPWYIWLGF	15	S	1206	1220
243	KWPWYIWLGFIAGLI	15	S	1211	1225
244	IWLGFIAGLIAIVMV	15	S	1216	1230
245	IAGLIAIVMVTIMLC	15	S	1221	1235
246	AIVMVTIMLCCMTSC	15	S	1226	1240
247	TIMLCCMTSCCSCLK	15	S	1231	1245
248	CMTSCCSCLKGCCSC	15	S	1236	1250
249	CSCLKGCCSCGSCCK	15	S	1241	1255
250	GCCSCGSCCKFDEDD	15	S	1246	1260
251	GSCCKFDEDDSEPVL	15	S	1251	1265
252	FDEDDSEPVLKGVKL	15	S	1256	1270
253	DDSEPVLKGVKLHYT	15	S	1261	1275

Supplementary Table 2. Non-spike peptides

Peptide	Description	Len	Antigen	Start	End	Dominant
1	FYVYSRVKNLNSSRV	15	E	56	70	Yes
2	QFAYANRNRFLYIIK	15	M	36	50	Yes
3	VLAAVYRINWITGGI	15	M	66	80	Yes
4	YRINWITGGIAIAMA	15	M	71	85	Yes
5	CLVGLMWLSYFIASF	15	M	86	100	Yes
6	MWLSYFIASFRLFAR	15	M	91	105	Yes
7	TNILLNVPLHGTILT	15	M	116	130	Yes
8	SELVIGAVILRGHLR	15	M	136	150	Yes
9	GAVILRGHLRIAGHHLGR	18	M	141	158	Yes
10	LRGHLRIAGHHLGRC	15	M	145	159	Yes
11	RGHLRIAGHHLGRCD	15	M	146	160	Yes
12	IAGHHLGRCDIKDLP	15	M	151	165	Yes
13	LGRCDIKDLPKEITV	15	M	156	170	Yes
14	IKDLPKEITVATSRT	15	M	161	175	Yes
15	KEITVATSRTLSYYK	15	M	166	180	Yes
16	TSRTLSYYKLGASQRVA	17	M	172	188	Yes
17	SRTLSYYKLGASQRV	15	M	173	187	Yes
18	LSYYKLGASQRVAGD	15	M	176	190	Yes
19	SYYKLGASQRVAGDS	15	M	177	191	Yes
20	LGASQRVAGDSGFAA	15	M	181	195	Yes
21	SGFAAYSRYRIGNYK	15	M	191	205	Yes
22	ASWFTALTQHGKEDL	15	N	50	64	Yes
23	SWFTALTQHGKEDLK	15	N	51	65	Yes
24	DDQIGYYRRATRRIR	15	N	81	95	Yes
25	IGYYRRATRRIRGGD	15	N	84	98	Yes
26	YYRRATRRIRGGDGK	15	N	86	100	Yes
27	MKDLSPRWYFYYLGT	15	N	101	115	Yes
28	RWYFYYLGTGPEAGL	15	N	107	121	Yes
29	NKDGIIWVATEGALN	15	N	126	140	Yes
30	KDGIIWVATEGALNT	15	N	127	141	Yes
31	AIVLQLPQGTTLPKG	15	N	156	170	Yes
32	AGNGGDAALALLLLD	15	N	211	225	Yes
33	DAALALLLLDRLNQL	15	N	216	230	Yes
34	LLLLDRLNQLESKMS	15	N	221	235	Yes
35	AAEASKKPRQKRTAT	15	N	251	265	Yes
36	KKPRQKRTATKAYNV	15	N	256	270	Yes
37	KRTATKAYNVTQAFG	15	N	261	275	Yes
38	KAYNVTQAFGRRGPE	15	N	266	280	Yes
39	LIRQGTDYKHWPQIA	15	N	291	305	Yes
40	YKHWPQIAQFAPSAS	15	N	298	312	Yes
41	WPQIAQFAPSASAFF	15	N	301	315	Yes
42	ASAFFGMSRIGMEVT	15	N	311	325	Yes
43	GMEVTPSGTWLTYTGAI KLD	20	N	321	340	Yes
44	PSGTWLTYTGAIKLD	15	N	326	340	Yes
45	GTWLTYTGAIKLDDK	15	N	328	342	Yes
46	FKDQVILLNKHIDAY	15	N	346	360	Yes
47	ILLNKHIDAYKTFPP	15	N	351	365	Yes
48	LMIERFVSLAIDAYP	15	NSP12	854	868	Yes
49	LDDFVEIIKSQDLSV	15	NSP15	299	313	Yes
50	KVTFFPDLNGDVVAI	15	NSP3	1138	1152	Yes
51	KHFYWFFSNYLKRRV	15	NSP4	388	402	Yes
52	NRYFRLTLGVYDYLV	15	NSP6	232	246	Yes
53	INVFAFPFTIYSLLL	15	ORF10	4	18	Yes
54	FMRIFTIGTVTLKQG	15	ORF3a	4	18	Yes
55	KKRWQLALSKGVHFV	15	ORF3a	66	80	Yes
56	LYLYALVYFLQSINF	15	ORF3a	106	120	Yes
57	QSINFVRIIMRLWLC	15	ORF3a	116	130	Yes
58	IWNLDYIINLIIKNL	15	ORF6	26	40	Yes
59	QEEVQELYSPIFLIV	15	ORF7a	90	104	Yes
60	TQHQPYVVDDPCPIH	15	ORF8	26	40	Yes
61	YVVDDPCPIHFYSKW	15	ORF8	31	45	Yes
62	PCPIHFYSKWYIRVG	15	ORF8	36	50	Yes
63	FYSKWYIRVGARKSA	15	ORF8	41	55	Yes
64	SKWYIRVGARKSAPL	15	ORF8	43	57	Yes
65	YIRVGARKSAPLIEL	15	ORF8	46	60	Yes
66	IGNYTVSCLPFTINC	15	ORF8	76	90	Yes
67	FTINCQEPKLGSLVV	15	ORF8	86	100	Yes
68	GSLVVRCSFYEDFLE	15	ORF8	96	110	Yes
69	RCSFYEDFLEYHDVR	15	ORF8	101	115	Yes

Peptide	Description	Len	Antigen	Start	End	Dominant
1	SEETGTLIVNSVLLF	15	E	6	20	No
2	FLLVTLAILTALRLC	15	E	26	40	No
3	LAILTALRLCAYCCN	15	E	31	45	No
4	EELKKLLEQWNLVIG	15	M	11	25	No
5	LLEQWNLVIGFLFLT	15	M	16	30	No
6	NLVIGFLFLTWICLL	15	M	21	35	No
7	FLFLTWICLLQFAYA	15	M	26	40	No
8	WICLLQFAYANRNRF	15	M	31	45	No
9	NRNRFLYIIKLIFLW	15	M	41	55	No
10	LYIIKLIFLWLLWPV	15	M	46	60	No
11	LIFLWLLWPVTLACF	15	M	51	65	No
12	LLWPVTLACFVLAAV	15	M	56	70	No
13	ITGGIAIAMACLVGL	15	M	76	90	No
14	AIAMACLVGLMWLSY	15	M	81	95	No
15	FIASFRLFARTRSMW	15	M	96	110	No
16	RLFARTRSMWSFNPE	15	M	101	115	No
17	TRSMWSFNPETNILL	15	M	106	120	No
18	SFNPETNILLNVPLH	15	M	111	125	No
19	NVPLHGTILTRPLLE	15	M	121	135	No
20	GTILTRPLLESELVI	15	M	126	140	No
21	RPLLESELVIGAVIL	15	M	131	145	No
22	GAVILRGHLRIAGHH	15	M	141	155	No
23	LRIAGHHLGRCDIKD	15	M	149	163	No
24	ATSRTLSYYKLGASQ	15	M	171	185	No
25	RVAGDSGFAAYSRYR	15	M	186	200	No
26	YSRYRIGNYKLNTDH	15	M	196	210	No
27	IGNYKLNTDHSSSSD	15	M	201	215	No
28	LNTDHSSSSDNIALL	15	M	206	220	No
29	PQNQRNAPRITFGGP	15	N	6	20	No
30	NAPRITFGGPSDSTG	15	N	11	25	No
31	TFGGPSDSTGSNQNG	15	N	16	30	No
32	LTQHGKEDLKFPRGQ	15	N	56	70	No
33	FPRGQGVPINTNSSP	15	N	66	80	No
34	GVPINTNSSPDDQIG	15	N	71	85	No
35	TRRIRGGDGKMKDLS	15	N	91	105	No
36	GGDGKMKDLSPRWYF	15	N	96	110	No
37	MKDLSPRWYFYYLGT GPEAG	20	N	101	120	No
38	PRWYFYYLGTGPEAG	15	N	106	120	No
39	LPYGANKDGIIWVAT	15	N	121	135	No
40	IWVATEGALNTPKDH	15	N	131	145	No
41	EGALNTPKDHIGTRN	15	N	136	150	No
42	GKGQQQQGQTVTKKS	15	N	236	250	No
43	KPRQKRTATKAYNVT	15	N	257	271	No
44	TQAFGRRGPEQTQGN	15	N	271	285	No
45	RRGPEQTQGNFGDQE	15	N	276	290	No
46	FGDQELIRQGTDYKH	15	N	286	300	No
47	TDYKHWPQIAQFAPS	15	N	296	310	No
48	QFAPSASAFFGMSRI	15	N	306	320	No
49	AFFGMSRIGMEVTPS	15	N	313	327	No
50	GMEVTPSGTWLTYTG	15	N	321	335	No
51	TWLTYTGAIKLDDKDP NF	18	N	329	346	No
52	LTYTGAIKLDDKDPN	15	N	331	345	No
53	AIKLDDKDPNFKDQV	15	N	336	350	No
54	PNFKDQVILLNKHIDAY K	18	N	344	361	No
55	HIDAYKTFPPTEPKK	15	N	356	370	No
56	QKKQQTVTLLPAADL	15	N	386	400	No
57	KQQTVTLLPAADLDF	16	N	388	403	No
58	TVTLLPAADLDDFSK	15	N	391	405	No
59	AADLDDFSKQLQQSM	15	N	397	411	No
60	VLSFCAFAVDAAKAY	15	NSP10	13	27	No
61	PDILRVYANLGERVR	15	NSP12	169	183	No
62	SLLMPILTLTRALTA	15	NSP12	239	253	No
63	HCANFNVLFSTVFPP	15	NSP12	309	323	No
64	NVLFSTVFPPTSFGP	15	NSP12	314	328	No
65	QDALFAYTKRNVIPT	15	NSP12	524	538	No
66	KLLKSIAATRGATVV	15	NSP12	574	588	No
67	IAATRGATVVIGTSK	15	NSP12	579	593	No
68	YPKCDRAMPNMLRIM	15	NSP12	619	633	No
69	RAMPNMLRIMASLVL	15	NSP12	624	638	No

Peptide	Description	Len	Antigen	Start	End	Dominant
70	SHRFYRLANECAQVL	15	NSP12	649	663	No
71	SEMVMCGGSLYVKPG	15	NSP12	664	678	No
72	FNICQAVTANVNALL	15	NSP12	694	708	No
73	AVTANVNALLSTDGN	15	NSP12	699	713	No
74	EFYAYLRKHFSMMIL	15	NSP12	744	758	No
75	LRKHFSMMILSDDAV	15	NSP12	749	763	No
76	GLVASIKNFKSVLYY	15	NSP12	774	788	No
77	KTDGTLMIERFVSLA	15	NSP12	849	863	No
78	FVSLAIDAYPLTKHP	15	NSP12	859	873	No
79	IDAYPLTKHPNQEYA	15	NSP12	864	878	No
80	DVFHLYLQYIRKLHD	15	NSP12	879	893	No
81	TSHKLVLSVNPYVCN	15	NSP13	37	51	No
82	ELHLSWEVGKPRPPL	15	NSP13	162	176	No
83	PRPPLNRNYVFTGYR	15	NSP13	172	186	No
84	FTGYRVTKNSKVQIG	15	NSP13	182	196	No
85	VTKNSKVQIGEYTFE	15	NSP13	187	201	No
86	VNARLRAKHYVYIGD	15	NSP13	387	401	No
87	ISPYNSQNAVASKIL	15	NSP13	512	526	No
88	NVNRFNVAITRAKVG	15	NSP13	557	571	No
89	NMFITREEAIRHVRA	15	NSP14	71	85	No
90	REEAIRHVRAWIGFD	15	NSP14	76	90	No
91	PLMYKGLPWNVVRIK	15	NSP14	151	165	No
92	EIIKSQDLSVVSKVV	15	NSP15	304	318	No
93	TQLCQYLNTLTLAVP	15	NSP16	48	62	No
94	AVMSLKEGQINDMIL	15	NSP16	258	272	No
95	KEGQINDMILSLLSK	15	NSP16	263	277	No
96	RENNRVVISSDVLVN	15	NSP16	283	297	No
97	PLNSIIKTIQPRVEK	15	NSP2	96	110	No
98	EEIAIILASFSASTS	15	NSP2	291	305	No
99	SPLYAFASEAARVVR	15	NSP2	351	365	No
100	AITILDGISQYSLRL	15	NSP2	386	400	No
101	QTFFKLVNKFLALCA	15	NSP2	496	510	No
102	GETFVTHSKGLYRKC	15	NSP2	526	540	No
103	ADAVIKTLQPVSELL	15	NSP3	58	72	No
104	ESDDYIATNGPLKVG	15	NSP3	268	282	No
105	IATNGPLKVGGSCVL	15	NSP3	273	287	No
106	SGHNLAKHCLHVVGP	15	NSP3	288	302	No
107	NLYDKLVSSFLEMKS	15	NSP3	363	377	No
108	ENLLLYIDINGNLHP	15	NSP3	433	447	No
109	KSAFYILPSIISNEK	15	NSP3	532	546	No
110	RFYFYTSKTTVASLI	15	NSP3	603	617	No
111	EAARYMRSLKVPATV	15	NSP3	643	657	No
112	LPNDDTLRVEAFEYY	15	NSP3	803	817	No
113	TLRVEAFEYYHTTDP	15	NSP3	808	822	No
114	HTTDPSFLGRYMSAL	15	NSP3	818	832	No
115	SFLGRYMSALNHTKK	15	NSP3	823	837	No
116	YMSALNHTKKWKYPQ	15	NSP3	828	842	No
117	NHTKKWKYPQVNGLT	15	NSP3	833	847	No
118	ESPFVMMSAPPAQYE	15	NSP3	983	997	No
119	YCIDGALLTKSSEYK	15	NSP3	1028	1042	No
120	DNFKFVCDNIKFADD	15	NSP3	1108	1122	No
121	LNQLTGYKKPASREL	15	NSP3	1123	1137	No
122	GYKKPASRELKVTFF	15	NSP3	1128	1142	No
123	ASRELKVTFFPDLNG	15	NSP3	1133	1147	No
124	PDLNGDVVAIDYKHY	15	NSP3	1143	1157	No
125	TPSFKKGAKLLHKPI	15	NSP3	1158	1172	No
126	VWHVNNATNKATYKP	15	NSP3	1173	1187	No
127	MAAYVDNSSLTIKKP	15	NSP3	1278	1292	No
128	NELSRVLGLKTLATH	15	NSP3	1293	1307	No
129	TFTRSTNSRIKASMP	15	NSP3	1363	1377	No
130	TNSRIKASMPTTIAK	15	NSP3	1368	1382	No
131	NTVKSVGKFCLEASF	15	NSP3	1383	1397	No
132	LEASFNYLKSPNFSK	15	NSP3	1393	1407	No
133	PNFSKLINIIIWFLL	15	NSP3	1403	1417	No
134	GSLIYSTAALGVLMS	15	NSP3	1423	1437	No
135	ISSFKWDLTAFGLVA	15	NSP3	1493	1507	No
136	WDLTAFGLVAEWFLA	15	NSP3	1498	1512	No
137	FGLVAEWFLAYILFT	15	NSP3	1503	1517	No
138	FDAYVNTFSSTFNVP	15	NSP3	1773	1787	No
139	SHNIALIWNVKDFMS	15	NSP3	1888	1902	No
140	KGGKIVNNWLKQLIK	15	NSP4	-2	12	No
141	LFVAAIFYLITPVHV	15	NSP4	18	32	No
142	AVITREVGFVVPGLP	15	NSP4	93	107	No
143	VPGLPGTILRTTNGD	15	NSP4	103	117	No
144	FLHFLPRVFSAVGNI	15	NSP4	118	132	No
145	DTRYVLMDGSIIQFP	15	NSP4	188	202	No
146	SIVAGGIVAIVVTCL	15	NSP4	283	297	No
147	FGEYSHVVAFNTLLF	15	NSP4	308	322	No
148	NTLLFLMSFTVLCLT	15	NSP4	318	332	No
149	PVYSFLPGVYSVIYL	15	NSP4	333	347	No
150	YLTFYLTNDVSFLAH	15	NSP4	348	362	No
151	SFLAHIQWMVMFTPL	15	NSP4	358	372	No
152	IQWMVMFTPLVPFWI	15	NSP4	363	377	No
153	MFTPLVPFWITIAYI	15	NSP4	368	382	No
154	TIAYIICISTKHFYW	15	NSP4	378	392	No
155	CTFLLNKEMYLKLRS	15	NSP4	418	432	No
156	LTQYNRYLALYNKYK	15	NSP4	438	452	No
157	RYLALYNKYKYFSGA	15	NSP4	443	457	No
158	YREAACCHLAKALND	15	NSP4	463	477	No
159	CCHLAKALNDFSNSG	15	NSP4	468	482	No
160	FSNSGSDVLYQPPQT	15	NSP4	478	492	No
161	SDVLYQPPQTSITSA	15	NSP4	483	497	No
162	NHNFLVQAGNVQLRV	15	NSP5	63	77	No
163	QNCVLKLKVDTANPK	15	NSP5	83	97	No
164	LLVLVQSTQWSLFFF	15	NSP6	22	36	No
165	SLFFFLYENAFLPFA	15	NSP6	32	46	No
166	LCLFLLPSLATVAYF	15	NSP6	67	81	No
167	TLVYKVYYGNALDQA	15	NSP6	147	161	No
168	DAFKLNIKLLGVGGK	15	NSP6	267	281	No
169	RVESSSKLWAQCVQL	15	NSP7	21	35	No
170	SKLWAQCVQLHNDIL	15	NSP7	26	40	No
171	VLKKLKKSLNVAKSE	15	NSP8	34	48	No
172	LIVTALRANSAVKLQ	15	NSP8	184	198	No
173	SDFVRATATIPIQAS	15	ORF3a	26	40	No
174	ALLAVFQSASKIITL	15	ORF3a	51	65	No
175	KIITLKKRWQLALSK	15	ORF3a	61	75	No
176	CNLLLLFVTVYSHLL	15	ORF3a	81	95	No
177	LVAAGLEAPFLYLYA	15	ORF3a	96	110	No
178	LEAPFLYLYALVYFL	15	ORF3a	101	115	No
179	LVYFLQSINFVRIIM	15	ORF3a	111	125	No
180	VRIIMRLWLCWKCRS	15	ORF3a	121	135	No
181	RLWLCWKCRSKNPLL	15	ORF3a	126	140	No
182	KNPLLYDANYFLCWH	15	ORF3a	136	150	No
183	YDANYFLCWHTNCYD	15	ORF3a	141	155	No
184	FLCWHTNCYDYCIPY	15	ORF3a	146	160	No
185	TNCYDYCIPYNSVTS	15	ORF3a	151	165	No
186	YFTSDYYQLYSTQLS	15	ORF3a	206	220	No
187	TDTGVEHVTFFIYNK	15	ORF3a	221	235	No
188	EHVTFFIYNKIVDEP	15	ORF3a	226	240	No
189	FIYNKIVDEPEEHVQ	15	ORF3a	231	245	No
190	GSSGVVNPVMEPIYD	15	ORF3a	251	265	No
191	MFHLVDFQVTIAEIL	15	ORF6	1	15	No
192	IAEILLIIMRTFKVS	15	ORF6	11	25	No
193	AEILLIIMRTFKVSI	15	ORF6	12	26	No
194	LIIMRTFKVSIWNLD	15	ORF6	16	30	No
195	TFKVSIWNLDYIINL	15	ORF6	21	35	No
196	YIINLIIKNLSKSLT	15	ORF6	31	45	No
197	MKIILFLALITLATC	15	ORF7a	1	15	No
198	IILFLALITLATCEL	15	ORF7a	3	17	No
199	DGVKHVYQLRARSVSPKL	18	ORF7a	69	86	No
200	VKHVYQLRARSVSPK	15	ORF7a	71	85	No
201	LYSPIFLIVAAIVFI	15	ORF7a	96	110	No
202	SPIFLIVAAIVFITL	15	ORF7a	98	112	No
203	DFYLCFLAFLLFLVL	15	ORF7b	8	22	No
204	MKFLVFLGIITTVAA	15	ORF8	1	15	No
205	FLGIITTVAAFHQEC	15	ORF8	6	20	No
206	TTVAAFHQECSLQSC	15	ORF8	11	25	No
207	FHQECSLQSCTQHQP	15	ORF8	16	30	No
208	SLQSCTQHQPYVVDD	15	ORF8	21	35	No
209	ARKSAPLIELCVDEA	15	ORF8	51	65	No
210	PLIELCVDEAGSKSP	15	ORF8	56	70	No
211	CVDEAGSKSPIQYID	15	ORF8	61	75	No
212	IQYIDIGNYTVSCLP	15	ORF8	71	85	No
213	QEPKLGSLVVRCSFY	15	ORF8	91	105	No
214	EDFLEYHDVRVVLDF	15	ORF8	106	120	No
215	DFLEYHDVRVVLDFI	15	ORF8	107	121	No

Supplementary Table 3. Percentage of memory T cells

Spike-specific memory T cells

Number of vaccinations	Subject #	CD4+ T helper cells Percentage out of AIM+ T cells			CD8+ cytotoxic T cells Percentage out of AIM+ T cells		
		**TEMRA**	**TEM**	**TCM**	**TEMRA**	**TEM**	**TCM**
**I**	10	0	91.1	8.9	0	0	0
**II or III**	1	0.3	74.7	24.2	13.7	83.0	3.3
	4	0	90.7	9.3	0	90.9	0
	5	0	89.2	10.4	0	0	0
	6	0	84.3	15.3	6.25	62.5	6.25
	7	0	75.4	23.7	35.3	23.5	5.9
	11	2.85	91.8	5.06	20.0	73.8	1.3
**≥ IV**	2	0.1	85.3	14.0	13.0	77.6	5.4
	3	0	67.6	31.5	0	100.0	0
	8	0	86.8	10.3	66.7	25.0	0
	9	12.1	74.6	8.98	65.0	17.5	0
	12	2.4	82.3	14.0	75.9	21.1	1.6

Non-spike-specific memory T cells

Number of vaccinations	Subject #	CD4+ T helper cells Percentage out of AIM+ T cells			CD8+ cytotoxic T cells Percentage out of AIM+ T cells		
		**TEMRA**	**TEM**	**TCM**	**TEMRA**	**TEM**	**TCM**
**I**	10	0.8	78.4	20.1	83.3	11.9	0
**II or III**	1	0.3	62.5	36.0	13.7	80.8	4.0
	4	0	78.3	21.6	0	78.6	21.4
	5	0	87.4	11.5	0	0	0
	6	0.5	82.4	16.1	0	91.7	0
	7	0	74.1	24.7	40.0	20.0	10.0
	11	3.15	90.3	5.41	29.0	71.0	0
**≥ IV**	2	0	84.9	13.0	14.0	56.0	10.0
	8	1.1	74.2	20.2	44.8	20.7	13.8
	9	10.4	75.6	8.5	51.5	12.1	9.1
